# Expectations of and perceived need for civil war in the USA: findings from a 2023 nationally representative survey

**DOI:** 10.1186/s40621-024-00521-5

**Published:** 2024-08-29

**Authors:** Garen J. Wintemute, Yueju Li, Bradley Velasquez, Andrew Crawford, Paul M. Reeping, Elizabeth A. Tomsich

**Affiliations:** 1 Department of Emergency Medicine, School of Medicine, University of California Davis, Sacramento, CA USA; 2 Violence Prevention Research Program, University of California Davis, Sacramento, CA USA; 3 California Firearm Violence Research Center, University of California Davis, Sacramento, CA USA; 4 Department of Public Health Sciences, School of Medicine, University of California Davis, Sacramento, CA USA

**Keywords:** Civil war, Political violence, Firearms, Firearm violence, Violence and society, Racism, Domestic violent extremism, White supremacy, Christian nationalism, Militia movement, Boogaloo movement, Proud boys, Oath keepers, Three percenters, QAnon

## Abstract

**Background:**

Surveys have found concerningly high levels of agreement that the United States will experience civil war soon. This study assesses variation in expectation of and perceived need for civil war with respondent sociopolitical characteristics, beliefs, firearm ownership, and willingness to engage in political violence.

**Methods:**

Findings are from Wave 2 of a nationally representative annual longitudinal survey of members of the Ipsos KnowledgePanel, conducted May 18–June 8, 2023. All respondents to 2022’s Wave 1 who remained in KnowledgePanel were invited to participate. Outcomes are expressed as weighted proportions and adjusted prevalence differences, with p-values adjusted for the false discovery rate and reported as q-values.

**Results:**

The completion rate was 84.2%; there were 9385 respondents. After weighting, half the sample was female (50.7%, 95% CI 49.4%, 52.1%); the weighted mean (± standard deviation) age was 48.5 (25.9) years. Approximately 1 respondent in 20 (5.7%, 95% CI 5.1%, 6.4%) agreed strongly or very strongly that “in the next few years, there will be civil war in the United States.” About 1 in 25 (3.8%, 95% CI 3.2%, 4.4%), and nearly 40% (38.4%, 95% CI 32.3%, 44.5%) of those who strongly or very strongly agreed that civil war was coming, also agreed strongly or very strongly that “the United States needs a civil war to set things right.” Expectation of and perceived need for civil war were higher among subsets of respondents who in Wave 1 were more willing than others to commit political violence, including MAGA Republicans, persons in strong agreement with racist beliefs or statements of the potential need for violence to effect social change, persons who strongly approved of specified extreme right-wing political organizations and movements, firearm owners who purchased firearms in 2020 or later, and firearm owners who carried firearms in public all or nearly all the time.

**Conclusions:**

In 2023, the expectation that civil war was likely and the belief that it was needed were uncommon but were higher among subsets of the population that had previously been associated with greater willingness to commit political violence. These findings can help guide prevention efforts.

**Supplementary Information:**

The online version contains supplementary material available at 10.1186/s40621-024-00521-5.

## Background

Recent concerns about political violence in the United States (USA) extend to the possibility of widespread civil conflict (Walter 2022; Kleinfeld 2023; Gale and West 2021; Simon and Stevenson 2023; Walter et al. 2022). Days after the January 6, 2021 assault on the Capitol, a public opinion poll found that 46% of likely voters thought “another civil war” was “likely” (Zogby. 2021). In mid-2022, Wave 1 of our nationally representative longitudinal survey on political violence in the US found that 13.7% of adults strongly or very strongly agreed, and another 36.4% somewhat agreed, with the statement that “in the next few years, there will be civil war in the United States” (Wintemute et al. 2023a).

This concerning finding led us to expand our investigation into expectations of and attitudes toward a possible civil war in Wave 2 of the survey, which was conducted in May–June 2023 (Wintemute et al. 2024a). We again asked respondents whether they thought civil war was coming and asked them to predict what form such a conflict might take. We also asked how much they agreed with the assertion that “the United States needs a civil war to set things right.”

It would be difficult to overstate the consequences of large-scale political violence, whatever form it took. Thousands of people could be killed or injured. At minimum, health systems face the likelihood that mass casualty events arising from political violence would come with a unique level of risk that patients would be armed and that other armed persons would seek access to the facilities providing care—perhaps by force. The conflict that produced the mass casualties could break out again at those facilities. Diminished capacity of health systems could lead to increased mortality and morbidity from other causes. But the greatest damage would almost certainly be to other vital physical and social infrastructures, from power grids to government’s ability to govern to social cohesion itself (Walter 2022; National Security Council 2021; Sousa 2013), producing adverse effects that could persist for decades.

In this study we assess expectations of and support for civil war in the US among the 9385 participants in Wave 2 of the survey, including variation in those measures with participants’ sociopolitical characteristics, firearm ownership and use, stated willingness to commit political violence, and other key attributes, based largely on their responses to items presented in Wave 1 (Wintemute et al. 2023a, 2024b, 2024c, 2023b, 2022b). Better understanding the factors that influence support for large-scale civil conflict may play an important role in developing effective prevention measures.

## Methods

Methods for Wave 2 of this longitudinal survey closely followed those for Wave 1 (Wintemute et al. 2023a). Wave 2 was designed by the authors and administered online in English and Spanish from May 18 to June 8, 2023 by the survey research firm Ipsos (2024). The study was reviewed by the University of California Davis Institutional Review Board (protocol 187,125: exempt from full review, category 2, survey research). The IRB waived a requirement for written or verbal consent. Before participants accessed the questionnaire, they were provided informed consent language that concluded, “[by] continuing, you are agreeing to participate in this study.” The study is reported following American Association for Public Opinion Research guidelines (2021).

### Participants

Participants for Wave 1 were drawn from the Ipsos KnowledgePanel, an online research panel that has been widely used in population-based research on violence and firearm ownership (Kravitz-Wirtz et al. 2021; Wintemute et al. 2022a; Schleimer et al. 2020; Miller et al. 2022; Miller and Azrael 2022; Salhi et al. 2019). To establish a nationally representative panel, KnowledgePanel members are recruited on an ongoing basis through address-based probability sampling using data from the US Postal Service’s Delivery Sequence File (Ipsos 2015; Ipsos 2020). Recruitment into KnowledgePanel involves repeated contact attempts, if necessary, by mail and telephone. Recruited adults in households without internet access are provided a web-enabled device and free internet service, and a modest, primarily points-based incentive program seeks to encourage participation and promote participants’ retention in KnowledgePanel over time (Ipsos 2015; Ipsos 2020).

A probability-proportional-to-size procedure was used to select a study-specific sample for Wave 1. All panel members who were aged 18 years and older were eligible for selection. Invitations were sent by e-mail; automatic reminders were delivered to non-respondents by e-mail and telephone beginning 3 days later (Ipsos 2015; Ipsos 2020).

The Wave 1 survey was conducted May 13 to June 2, 2022. It included a main sample, which had a completion rate of 53% and provided the study population for our initial report (Wintemute et al. 2023a), and oversamples of firearm owners, transgender people, combat veterans, and California residents that were recruited to ensure adequate statistical power for planned analyses. Compared with main sample nonrespondents, main sample respondents were older and more frequently white, non-Hispanic; were more often married; had higher education and income; and were less likely to be working (Wintemute et al. 2023a).

Including the main sample and oversamples, Wave 1 comprised 12,947 respondents. Invitations to participate in Wave 2 were sent to the 11,140 Wave 1 respondents (86% of the 12,947) who remained active members of KnowledgePanel on Wave 2’s launch date. (The remaining 1807 Wave 1 respondents had left the cohort through normal attrition.)

A final Wave 2 survey weight variable provided by Ipsos adjusted for the initial probability of selection into KnowledgePanel and for survey-specific nonresponse and over- or under-coverage using design weights with post-stratification raking ratio adjustments. As with the 2022 sample, the weighted 2023 sample is designed to be statistically representative of the noninstitutionalized adult population of the US as reflected in the 2021 March supplement of the Current Population Survey (Ipsos 2015; Ipsos 2020).

### Measures

Sociodemographic data were collected by Ipsos from profiles created and maintained by KnowledgePanel members. Our primary measures of interest concerned respondents’ perceptions of the likelihood, need for, and probable form of civil war in the US. Participants were asked their “view of what a second civil war might look like,” with response options “like the first Civil War…with opposing armies and large battles” and “like an insurgency or guerrilla war, with small groups attacking specific targets or people.” They were then asked about their agreement with the following statements: “In the next few years, there will be civil war in the United States,” and “the United States needs a civil war to set things right.”

We assessed associations between responses to those items and survey items from Wave 1 or Wave 2 that covered 5 broad domains: political party affiliation and political ideology, beliefs about race and ethnicity and American society, beliefs about the potential need for violence to effect social change in the US, firearm ownership and use, and approval of eight extreme right-wing political organizations and movements: the Proud Boys, the Oath Keepers, the Three Percenters, QAnon, the Christian Nationalist movement, the white supremacy movement, the militia movement, and the Boogaloo movement. Further information on the construction of these measures has been published previously (Wintemute et al. 2023a, 2024b, 2024c, 2023b, 2022a) and is provided in Additional File 1, as are the full text of all questions reported on here and sources for questions from surveys by other investigators.

### Implementation

Ipsos translated the questionnaire into Spanish, and interpreting services staff at UC Davis Medical Center reviewed the translation. Thirty-three KnowledgePanel members participated in a pretest of the English language version that was administered May 5–9, 2023.

Respondents were randomized 1:1 to receive response options in order from either negative to positive valence (example: from ‘do not agree’ to ‘strongly agree’) or the reverse throughout the questionnaire. Where a question presented multiple statements for respondents to consider, the order in which those statements were presented was randomized unless ordering was necessary. Logic-driving questions (those to which responses might invoke a skip pattern) included non-response prompts.

We employed unipolar response arrays without a neutral midpoint (e.g., do not agree, somewhat agree, strongly agree, very strongly agree). The literature is not in agreement on whether such midpoints should be included (Chyung et al. 2017; Westwood et al. 2022). We were persuaded by the studies reviewed by Chyung et al. (2017), which suggest that such midpoints allow respondents to choose “a minimally acceptable response as soon as it is found, instead of putting effort to find an optimal response,” a behavior known as satisficing. According to those authors, satisficing is particularly common when respondents are uncomfortable with the topics of the survey or under social desirability pressures; both conditions apply here.

### Statistical analysis

Analyses were conducted using SAS version 9.4 (SAS Institute, Inc., Cary, NC). To generate prevalence estimates, we calculated weighted percentages and 95% confidence intervals (CI) using PROC SURVEYFREQ. A Spearman correlation coefficient was calculated using PROC CORR.

To compute adjusted prevalence differences and 95% CIs, we defined outcomes dichotomously and used PROC SURVEYREG, employing robust standard errors to correct for design effects and heteroskedasticity in binary outcomes. We considered several models (see Additional File 1), choosing the final model based on concordance with theory, findings from prior research, and fit statistics. That model included age, race and ethnicity, gender, education, income, Census division, and rurality.

P-values were corrected for multiple comparisons by controlling the false discovery rate using the Benjamini–Hochberg method (Benjamini and Hochberg 1995). The resulting values are known as FDR-adjusted (or FDR-corrected) *p*-values or as q-values (Storey 2003); we employ the latter term here. Q-values represent the probability that the given difference would be a false discovery; they represent the expected proportion of “false positives” that would be seen among the collection of all differences whose q-values were at or below the given q-value.

## Results

Of 11,140 panel members invited to participate, 9385 completed the survey, yielding an 84.2% completion rate. The median survey completion time was 25 min (interquartile range, 18.6 min). Item non-response in this analysis ranged from 0.4 to 5.7%; only 2 items had non-response percentages above 3.0% (see Additional File 1).

After weighting, half of the respondents (50.7%, 95% CI 49.4%, 52.1%) were female; 62.7% (95% CI 61.2%, 64.1%) were white, non-Hispanic (Table S1). The weighted mean (SD) respondent age was 48.5 (25.9) years. Table S2 presents unweighted sociodemographic characteristics for respondents and nonrespondents.

A large majority of respondents (83.1%, 95% CI 82.0%, 84.3%) believed that, were a civil war to occur, it would take the form of “an insurgency or guerrilla war” and not involve “opposing armies and large battles” (Table 1). Approximately 1 respondent in 20 (5.7%, 95% CI 5.1%, 6.4%) agreed strongly or very strongly with the proposition that “in the next few years, there will be civil war in the United States,” and about 1 in 25 (3.8%, 95% CI 3.2%, 4.4%) agreed strongly or very strongly that “the United States needs a civil war to set things right” (Table 1). Responses to these 2 items were correlated (Spearman correlation coefficient = 0.48); among respondents who strongly or very strongly agreed that civil war was coming, 38.4% (95% CI 32.3%, 44.5%) strongly or very strongly agreed that it was needed (Table 2).

**Table 1:** Expectations of and perceived need for civil war in the United States

Query and response	Unweighted n	Weighted % (95% CI)
Which of the following comes closer to your view of what a second civil war might look like?*		
Like an insurgency or guerrilla war, with small groups attacking specific targets or people	8171	83.1 (82.0, 84.3)
Like the first Civil War in the United States, with opposing armies and large battles	753	11.1 (10.1, 12.1)
Refused	461	5.7 (5.1, 6.4)
*How much do you agree or disagree with each of the following statements?*^†^		
In the next few years, there will be civil war in the United States		
Do not agree	6167	63.2 (61.9, 64.6)
Somewhat agree	2576	28.3 (27.1, 29.6)
Strongly or very strongly agree	480	5.7 (5.1, 6.4)
Refused	162	2.7 (2.2, 3.2)
The United States needs a civil war to set things right		
Do not agree	8096	84.5 (83.4, 85.5)
Somewhat agree	851	9.4 (8.6, 10.3)
Strongly or very strongly agree	297	3.8 (3.2, 4.4)
Refused	141	2.3 (1.9, 2.8)

*Some people talk about a second civil war in the United States. Which of the following comes closer to your view of what a second civil war might look like? 1. A second civil war would be like the first Civil War in the United States, with opposing armies and large battles. 2. A second civil war would be like an insurgency or guerrilla war, with small groups attacking specific targets or people

^†^Response options were do not agree, somewhat agree, strongly agree, very strongly agree. Findings are combined for the strongly and very strongly agree responses

**Table 2:** Relationship between expectations of and perceived need for civil war in the United States

In the Next Few Years, There Will Be Civil War in the United States*	The United States Needs a Civil War to Set Things Right*							
	Do Not Agree		Somewhat Agree		Strongly or Very Strongly Agree		Refused	
	Unweighted n	Weighted % (95% CI)	Unweighted n	Weighted % (95% CI)	Unweighted n	Weighted % (95% CI)	Unweighted n	Weighted % (95% CI)
Do not agree	5990	97.2 (96.6, 97.7)	156	2.3 (1.8, 2.8)	18	0.4 (0.2, 0.7)	3	0.0 (0.0, 0.1)
Somewhat agree	1885	71.7 (69.2, 74.1)	588	23.6 (21.3, 25.9)	97	4.5 (3.2, 5.7)	6	0.2 (0.0, 0.5)
Strongly or very strongly agree	194	39.0 (33.0, 45.0)	104	21.8 (16.5, 27.1)	181	38.4 (32.3, 44.5)	1	0.8 (0.0, 2.2)
Refused	27	17.3 (9.5, 25.1)	3	1.1 (0.0, 2.5)	1	0.8 (0.0, 2.3)	131	80.8 (72.9, 88.8)

*How much do you agree or disagree with each of the following statements? 1. In the next few years, there will be civil war in the United States. 2. The United States needs a civil war to set things right. Response options were do not agree, somewhat agree, strongly agree, very strongly agree. Findings are combined for the strongly/very strongly agree responses

Respondents’ views of what form a future civil war might take were associated with their views of its likelihood and desirability (Table 3). Expectation of formal conflict involving “opposing armies and large battles” was more common among those who agreed strongly or very strongly that “in the next few years, there will be civil war in the United States” [27.2% (95% CI 21.5%, 32.9%)] than among those who disagreed [8.5% (95% CI 7.4%, 9.6%)]. Similarly, respondents who strongly or very strongly agreed that “the United States needs a civil war to set things right” were more likely than those who disagreed to predict formal conflict [32.2% (95% CI 24.4%, 39.9%) and 9.1% (95% CI 8.2%, 10.1%), respectively].

**Table 3:** Relationship between expectations of and perceived need for civil war and prediction of the form such a war might take

Query and response	Which of the Following Comes Closer to Your View of What a Second Civil War Might Look Like?^†^					
	Opposing Armies and Large Battles		Insurgency or Guerrilla War		Refused	
	Unweighted n	Weighted % (95% CI)	Unweighted n	Weighted % (95% CI)	Unweighted n	Weighted % (95% CI)
How much do you agree or disagree with each of the following statements?*						
In the next few years, there will be civil war in the United States						
Do not agree	360	8.5 (7.4, 9.6)	5535	86.8 (85.5, 88.0)	272	4.7 (4.0, 5.5)
Somewhat agree	288	14.4 (12.3, 16.4)	2221	83.2 (81.1, 85.3)	67	2.4 (1.7, 3.2)
Strongly or very strongly agree	102	27.2 (21.5, 32.9)	371	71.7 (66.0, 77.5)	7	1.1 (0.0, 2.3)
Refused	3	4.2 (0.0, 9.5)	44	22.6 (14.9, 30.3)	115	73.2 (64.5, 81.8)
The United States needs a civil war to set things right						
Do not agree	530	9.1 (8.2, 10.1)	7329	86.5 (85.4, 87.6)	327	4.3 (3.7, 5.0)
Somewhat agree	136	22.1 (17.9, 26.4)	694	75.8 (71.5, 80.1)	21	2.0 (0.9, 3.2)
Strongly or very strongly agree	84	32.2 (24.4, 39.9)	208	66.8 (59.0, 74.5)	5	1.1 (0.0, 2.3)
Refused	3	4.1 (0.0, 10.0)	30	16.8 (9.7, 23.9)	108	79.1 (70.5, 87.7)

^*^Response options were do not agree, somewhat agree, strongly agree, very strongly agree. Findings are combined for the strongly/very strongly agree responses

^†^Some people talk about a second civil war in the United States. Which of the following comes closer to your view of what a second civil war might look like? 1. A second civil war would be like the first Civil War in the United States, with opposing armies and large battles. 2. A second civil war would be like an insurgency or guerrilla war, with small groups attacking specific targets or people

### Variation with sociodemographic characteristics, beliefs, and firearm ownership

The expectation that civil war was coming and the belief that it was needed were higher among strong Republicans, MAGA Republicans, non-Republican members of the MAGA movement, and extreme conservatives, relative to their respective comparison groups (Table 4, Tables S3–S5).

**Table 4:** Summary of findings on expectations of and perceived need for civil war for subgroups of respondents

Characteristic	"In the next few years, there will be civil war in the United States."*	"The United States needs a civil war to set things right."*
	Adjusted prevalence difference (95% CI; q-value)	Adjusted prevalence difference (95% CI; q-value)
Political party affiliation		
Strong Democrat	-1.1 (-3.5,1.3; 0.57)	-1.3 (-3.2, 0.7; 0.45)
Not very strong Democrat	-1.2 (-3.9, 1.5; 0.57)	-1.6 (-3.8, 0.6; 0.44)
Undecided/Independent/Other/Leans	Referent	Referent
Not very strong Republican	-1.2 (-3.6, 1.3; 0.57)	-0.6 (-2.8, 1.6; 0.75)
Strong Republican	6.0 (3.0, 8.9; 0.001)	4.4 (1.8, 6.9; 0.006)
MAGA status		
MAGA Republican	7.4 (4.1, 9.8; < 0.001)	6.3 (3.9, 8.6; < 0.001)
Other Republican	2.1 (0.5, 3.7; 0.01)	1.8 (0.4, 3.2; 0.01)
Non-Republican, MAGA movement	12.3 (4.7, 19.9; 0.003)	7.7 (1.8, 13.5; 0.01)
Non-Republican, not MAGA movement	Referent	Referent
Political ideology		
Extremely liberal	0.6 (-3.0, 4.2; 0.79)	0.9 (-2.2, 4.0; 0.75)
Liberal	-2.1 (-4.1, -0.2; 0.10)	-1.0 (-2.4, 0.4; 0.31)
Slightly liberal	-2.1 (-4.3, 0.1; 0.15)	0.7 (-2.0, 3.3; 0.75)
Moderate/Middle of the Road	Referent	Referent
Slightly conservative	-0.3 (-2.7, 2.0; 0.79)	1.1 (-0.9, 3.1; 0.44)
Conservative	1.1 (-1.0, 3.2; 0.44)	1.2 (-0.6, 2.9; 0.35)
Extremely conservative	5.5 (1.6, 9.4; 0.05)	5.5 (2.0, 8.9; 0.03)
Beliefs about race and ethnicity		
Non-agreement	Referent	Referent
Weak agreement	-0.1 (-1.6, 1.5; 0.94)	-0.4 (-1.3, 0.5; 0.48)
Moderate agreement	4.7 (2.8, 6.6; < 0.001)	4.4 (2.8, 5.9; < 0.001)
Strong agreement	7.1 (4.9, 9.3; < 0.001)	6.5 (4.6, 8.4; < 0.001)
Beliefs about violence to effect social change		
Non-agreement	Referent	Referent
Weak agreement	2.3 (0.9, 3.8; 0.002)	0.8 (0.0, 1.6; 0.04)
Moderate agreement	6.1 (4.0, 8.2; < 0.001)	5.7 (3.8, 7.7; < 0.001)
Strong agreement	28.3 (23.3, 33.2; < 0.001)	28.3 (23.4, 33.1; < 0.001)
Approval of organizations and movements		
Non-approval	Referent	Referent
Weak approval	3.7 (0.5, 6.9; 0.02)	4.6 (1.8, 7.4; 0.002)
Moderate approval	14.9 (6.4, 23.5; 0.001)	16.4 (8.0, 24.8; < 0.001)
Strong approval	33.5 (16.2, 50.8; < 0.001)	36.7 (19.7, 53.6; < 0.001)
Firearm ownership		
Non-owner, no firearms at home	Referent	Referent
Non-owner, firearms at home	-0.9 (-3.2, 1.3; 0.63)	0.3 (-1.8, 2.4; 0.78)
Owner	2.5 (1.0, 4.0; 0.005)	1.5 (0.3, 2.6; 0.04)
Type(s) of firearm owned		
Handgun only	Referent	Referent
Other	3.7 (-0.2, 7.6; 0.11)	3.6 (0.2, 7.0; 0.09)
Other rifle	1.2 (-1.4, 3.7; 0.42)	1.7 (-0.3, 3.7; 0.16)
Assault-type rifle	5.2 (2.0, 8.3; 0.01)	3.7 (1.0, 6.3; 0.03)
Recency of firearm purchase		
Purchases only 2019 or earlier	Referent	Referent
Purchases 2020 or later	2.5 (0.0, 4.9; 0.05)	3.9 (1.8, 5.9; 0.001)
Carrying in public in the past year		
Never or not often at all	Referent	Referent
Less than half, about half, or more than half the time	3.4 (0.4, 6.4; 0.04)	3.7 (1.1, 6.3; 0.01)
All or nearly all the time	7.9 (3.1, 12.6; 0.003)	6.9 (2.8, 11.0; 0.003)

Detailed findings for subgroups are in Tables S3–S13 (see Additional File 1)

*How much do you agree or disagree with each of the following statements? 1. In the next few years, there will be civil war in the United States. 2. The United States needs a civil war to set things right. Response options were do not agree, somewhat agree, strongly agree, very strongly agree

Adjusted models include age, race and ethnicity, gender, education, income, Census division, and rurality. Adjusted differences are for the strongly/very strongly agree comparison. Q-values represent the probability that the given difference would be a false discovery; they represent the expected proportion of “false positives” that would be seen among the collection of all differences whose q-values were at or below the given q-value

Expectation of and perceived need for civil war were also higher among respondents in strong or moderate agreement with racist beliefs compared with those in non-agreement (Table 4, Table S6). Expectation and perceived need were substantially higher (adjusted prevalence differences of approximately 28 percentage points) among respondents who strongly agreed with statements of the potential need for violence to effect social change than among those who did not agree (Table 4, Table S7), and among those who strongly approved of the specified extreme right-wing political organizations and movements as a group (adjusted prevalence differences of approximately 35 percentage points), compared with those who did not approve (Table 4, Table S8). These measures were also higher among respondents who strongly/very strongly approved of each of those organizations and movements individually, compared with those who did not approve (Table S9).

There were only small differences on expectation of and perceived need for civil war between firearm owners and non-owners without firearms at home (Table 4, Table S10). Among firearm owners, prevalences for both measures were higher among owners of assault-type rifles than among those who owned only handguns (Table 4, Table S11), among those who purchased firearms in 2020 or later than among those whose most recent purchase was in 2019 or earlier (Table 4, Table S12), and among those who carried firearms in public all or nearly all the time than among those who did so infrequently or never (Table 4, Table S13).

Across all subgroups, large majorities of respondents predicted that a future civil war would take the form of an insurgency (Tables S3–S13). However, there was greater expectation that future conflict would involve “opposing armies and large battles” among respondents who identified as MAGA Republicans or non-Republican members of the MAGA movement (Table S4), who agreed with racist beliefs (Table S6) or statements of the need for violence to effect social change (Table S7), and who supported extreme right-wing political organizations and movements (Tables S8, S9). There was no difference between firearm owners and non-owners (Table S10), but among owners, predictions of formal conflict were higher for owners of assault-type rifles (Table S11), recent purchasers (Table S12), and frequent carriers (Table S13).

### Variation with willingness to commit political violence and anticipated firearm use

Respondents who were very or completely willing to commit specified types of violence—to damage property, threaten a person, or kill a person—to advance political objectives were substantially more likely than unwilling respondents to agree strongly or very strongly that there would be civil war in the next few years and that civil war was needed (Table 5). The magnitude of the adjusted prevalence difference varied with the severity of the violence: between approximately 20 and 23 percentage points for those willing to commit property damage, compared with those who were not willing, but between approximately 28 and 36 percentage points for those willing versus those unwilling to commit threats or homicide. Respondents who were very or completely willing to commit violence also more frequently predicted (differences of approximately 13 to 15 percentage points) that a future civil conflict would involve “opposing armies and large battles.”

**Table 5:** Association between expectations and perceived need for civil war in the United States and personal willingness to engage in political violence, by type of violence

Query and response	Personal willingness to use violence to advance a political objective^‡^					
	Damage property					
	Not Willing		Somewhat Willing		Very/Completely Willing	
	Unweighted n	Weighted % (95% CI)	Unweighted n	Weighted % (95% CI)	Unweighted n	Weighted % (95% CI)
Which of the following comes closer to your view of what a second civil war might look like?*						
Like an insurgency or guerrilla war, with small groups attacking specific targets or people	5150	88.8 (87.6, 90.1)	664	86.1 (82.1, 90.0)	164	71.9 (63.4, 80.3)
Like the first Civil War in the United States, with opposing armies and large battles	465	11.2 (9.9, 12.4)	68	13.9 (10.0, 17.9)	49	28.1 (19.7, 36.6)
Adjusted prevalence difference (95% CI; q-value)^†^	Referent		1.7 (-2.2, 5.6; 0.40)		13.1 (4.6, 21.6; 0.005)	
How much do you agree or disagree with each of the following statements?^†^						
*In the next few years, there will be civil war in the United States*						
Do not agree	3797	63.1 (61.4, 64.9)	421	51.1 (46.1, 56.1)	90	39.8 (30.8, 48.7)
Somewhat agree	1722	31.5 (29.8, 33.1)	277	39.8 (34.9, 44.8)	76	33.7 (25.3, 42.0)
Strongly/very strongly agree	286	5.4 (4.5, 6.2)	53	9.1 (5.8, 12.3)	55	26.6 (18.4, 34.7)
Adjusted prevalence difference (95% CI; q-value)^§^	Referent		3.2 (-0.1, 6.5; 0.07)		19.6 (11.6, 27.5; < 0.001)	
*The United States needs a civil war to set things right*						
Do not agree	5051	86.5 (85.3, 87.7)	572	71.1 (66.2, 76.0)	125	50.5 (41.5, 59.5)
Somewhat agree	587	10.1 (9.1, 11.2)	131	20.9 (16.6, 25.3)	47	21.9 (14.1, 29.8)
Strongly/very strongly agree	176	3.4 (2.7, 4.0)	48	8.0 (4.8, 11.2)	50	27.6 (19.3, 35.8)
Adjusted prevalence difference (95% CI; q-value)^§^	Referent		4.0 (0.9, 7.1; 0.02)		22.8 (15.0, 30.7; < 0.001)	

Query and response	Personal willingness to use violence to advance a political objective^‡^					
	Threaten a person					
	Not Willing		Somewhat Willing		Very/Completely Willing	
	Unweighted n	Weighted % (95% CI)	Unweighted n	Weighted % (95% CI)	Unweighted n	Weighted % (95% CI)
Which of the following comes closer to your view of what a second civil war might look like?*						
Like an insurgency or guerrilla war, with small groups attacking specific targets or people	5189	89.1 (87.9, 90.4)	653	83.2 (78.7, 87.7)	127	71.0 (61.8, 80.2)
Like the first Civil War in the United States, with opposing armies and large battles	465	10.9 (9.6, 12.1)	73	16.8 (12.3, 21.3)	42	29.0 (19.8, 38.2)
Adjusted prevalence difference (95% CI; q-value)^†^	Referent		4.5 (0.2, 8.9; 0.04)		13.2 (4.0, 22.4; 0.008)	
How much do you agree or disagree with each of the following statements?^†^						
*In the next few years, there will be civil war in the United States*						
Do not agree	3856	63.4 (61.7, 65.1)	385	48.9 (43.7, 54.1)	64	34.9 (25.4, 44.4)
Somewhat agree	1726	31.6 (29.9, 33.2)	288	41.0 (35.8, 46.1)	57	30.0 (21.2, 38.9)
Strongly/very strongly agree	268	5.1 (4.3, 5.9)	69	10.1 (6.8, 13.4)	54	35.1 (25.3, 44.8)
Adjusted prevalence difference (95% CI; q-value)^§^	Referent		4.5 (1.0, 7.9; 0.01)		28.2 (18.7, 37.7; < 0.001)	
*The United States needs a civil war to set things right*						
Do not agree	5119	87 (85.8, 88.2)	544	67.8 (62.6, 72.9)	77	36.1 (26.8, 45.3)
Somewhat agree	579	9.9 (8.9, 11.0)	145	24.0 (19.2, 28.8)	41	22.9 (15.0, 30.9)
Strongly/very strongly agree	164	3.0 (2.4, 3.7)	52	8.2 (5.1, 11.4)	57	41.0 (30.9, 51.1)
Adjusted prevalence difference (95% CI; q-value)^§^	Referent		4.6 (1.4, 7.7; 0.008)		35.9 (26.3, 45.6; < 0.001)	

Query and response	Personal willingness to use violence to advance a political objective^‡^					
	Kill a person					
	Not willing		Somewhat willing		Very/completely Willing	
	Unweighted n	Weighted % (95% CI)	Unweighted n	Weighted % (95% CI)	Unweighted n	Weighted % (95% CI)
Which of the following comes closer to your view of what a second civil war might look like?*						
Like an insurgency or guerrilla war, with small groups attacking specific targets or people	5631	89.0 (87.8, 90.2)	238	74.6 (66.6, 82.5)	97	68.1 (57, 79.2)
Like the first Civil War in the United States, with opposing armies and large battles	501	11.0 (9.8, 12.2)	43	25.4 (17.5, 33.4)	38	31.9 (20.8, 43)
Adjusted prevalence difference (95% CI; q-value)^†^	Referent		11.6 (4.3, 18.9; 0.002)		15.4 (3.8, 27.0; 0.009)	
How much do you agree or disagree with each of the following statements?^†^						
*In the next few years, there will be civil war in the United States*						
Do not agree	4136	63.0 (61.4, 64.7)	127	35.0 (27.6, 42.4)	40	28.0 (16.8, 39.3)
Somewhat agree	1898	31.9 (30.3, 33.4)	120	44.3 (36.1, 52.4)	54	36.7 (25.7, 47.7)
Strongly/very strongly agree	304	5.1 (4.3, 5.9)	44	20.8 (13.3, 28.2)	43	35.3 (24.1, 46.4)
Adjusted prevalence difference (95% CI; q-value)^§^	Referent		14.8 (7.6, 22.0; < 0.001)		28.5 (17.4, 39.6; < 0.001)	
*The United States needs a civil war to set things right*						
Do not agree	5507	86.3 (85.1, 87.5)	175	48.5 (40.4, 56.7)	59	34.3 (23.6, 44.9)
Somewhat agree	648	10.3 (9.2, 11.3)	79	34.2 (26.1, 42.3)	36	29.1 (17.5, 40.6)
Strongly/very strongly agree	193	3.4 (2.7, 4.1)	36	17.3 (10.0, 24.5)	44	36.7 (25.7, 47.6)
Adjusted prevalence difference (95% CI; q-value)^§^	Referent		12.3 (5.1, 19.4; 0.001)		31.4 (20.5, 42.2; < 0.001)	

*Some people talk about a second civil war in the United States. Which of the following comes closer to your view of what a second civil war might look like? 1. A second civil war would be like the first Civil War in the United States, with opposing armies and large battles. 2. A second civil war would be like an insurgency or guerrilla war, with small groups attacking specific targets or people

^†^Adjusted prevalence difference is for the “like the first Civil War” response

^‡^In a situation where you think force or violence is justified to advance an important political objective, how willing would you personally be to use force or violence in each of these ways?

^§^Adjusted prevalence difference is for the strongly/very strongly agree response

Adjusted models include age, race and ethnicity, gender, education, income, Census division, and rurality. Adjusted differences are for the strongly/very strongly agree comparison. Q-values represent the probability that the given difference would be a false discovery; they represent the expected proportion of “false positives” that would be seen among the collection of all differences whose q-values were at or below the given q-value

Similarly, expectation and perceived need for civil war were more common among respondents who thought it very or extremely likely, as compared with respondents who thought it not likely, that they would use firearms in a future situation where they considered political violence justified (Table 6). The magnitude of the differences increased with the lethality of future uses of firearms that respondents considered to be very or extremely likely (Table 6): approximately 11 to 15 percentage points for “I will be armed with a gun,” 20 to 21 percentage points for “I will carry a gun openly,” and 37 to 41 percentage points for “I will shoot someone.” Again similarly, respondents who thought their own use of a firearm in future political violence was very or extremely likely were more likely than others to expect “opposing armies and large battles” in a future civil conflict.

**Table 6:** Association between future likelihood of firearm possession and use in a situation where political violence is perceived as justified and expectations and perceived need for civil war in the United States

Query and response	Anticipated Future Firearm Use When Political Violence Is Justified^‡^					
	I Will Be Armed with a Gun					
	Not LIkely		Somewhat LIkely		Very/Extremely Likely	
	Unweighted n	Weighted % (95% CI)	Unweighted n	Weighted % (95% CI)	Unweighted n	Weighted % (95% CI)
Which of the following comes closer to your view of what a second civil war might look like?*						
Like an insurgency or guerrilla war, with small groups attacking specific targets or people	6082	90.1 (89.0, 91.1)	1075	83.1 (79.5, 86.6)	954	79.6 (75.5, 83.6)
Like the first Civil War in the United States, with opposing armies and large battles	465	9.9 (8.9, 11.0)	141	16.9 (13.4, 20.5)	142	20.4 (16.4, 24.5)
Adjusted prevalence difference (95% CI; q-value)^†^	Referent		5.5 (2.1, 8.9; 0.002)		9.6 (5.6, 13.7; < 0.001)	
*How much do you agree or disagree with each of the following statements?*^*†*^						
In the next few years, there will be civil war in the United States						
Do not agree	4890	70.1 (68.6, 71.6)	704	51.3 (47.3, 55.3)	533	42.8 (38.7, 46.9)
Somewhat agree	1649	26.1 (24.7, 27.6)	459	39.7 (35.7, 43.6)	436	38.3 (34.3, 42.3)
Strongly/very strongly agree	222	3.8 (3.1, 4.4)	89	9.0 (6.5, 11.6)	164	18.9 (15.3, 22.5)
Adjusted prevalence difference (95% CI; q-value)^§^	Referent		4.6 (2.0, 7.3; 0.001)		14.7 (11.1, 18.3; < 0.001)	
*The United States needs a civil war to set things right*						
Do not agree	6246	90.8 (89.8, 91.8)	1006	76.7 (73.1, 80.3)	783	64.5 (60.3, 68.6)
Somewhat agree	415	6.8 (6.0, 7.7)	188	17.4 (14.2, 20.7)	233	21.5 (18.0, 24.9)
Strongly/very strongly agree	118	2.3 (1.8, 2.9)	61	5.9 (3.7, 8.0)	116	14.1 (10.8, 17.3)
Adjusted prevalence difference (95% CI; q-value)^§^	Referent		3.0 (0.8, 5.1; 0.006)		11.1 (7.8, 14.4; < 0.001)	

Query and response	Anticipated Future Firearm Use When Political Violence Is Justified^‡^					
	I Will Carry a Gun Openly, So That People Know I Am Armed					
	Not LIkely		Somewhat LIkely		Very/Extremely Likely	
	Unweighted n	Weighted % (95% CI)	Unweighted n	Weighted % (95% CI)	Unweighted n	Weighted % (95% CI)
Which of the following comes closer to your view of what a second civil war might look like?*						
Like an insurgency or guerrilla war, with small groups attacking specific targets or people	7117	89.9 (88.9, 90.9)	652	80.9 (76.3, 85.5)	338	68.9 (61.7, 76.1)
Like the first Civil War in the United States, with opposing armies and large battles	546	10.1 (9.1, 11.1)	104	19.1 (14.5, 23.7)	92	31.1 (23.9, 38.3)
Adjusted prevalence difference (95% CI; q-value)^†^	Referent		7.0 (2.5, 11.5; 0.002)		17.0 (9.9, 24.1; < 0.001)	
*How much do you agree or disagree with each of the following statements?*^*†*^						
In the next few years, there will be civil war in the United States						
Do not agree	5577	68.7 (67.3, 70.1)	384	43.2 (38.0, 48.4)	158	32.2 (25.6, 38.7)
Somewhat agree	2037	27.1 (25.8, 28.4)	329	43.7 (38.4, 48.9)	178	41.4 (34.5, 48.3)
Strongly/very strongly agree	302	4.2 (3.5, 4.8)	64	13.1 (8.9, 17.3)	108	26.5 (20.5, 32.4)
Adjusted prevalence difference (95% CI; q-value)^§^	Referent		7.8 (3.7, 12; < 0.001)		21.3 (15.3, 27.3; < 0.001)	
*The United States needs a civil war to set things right*						
Do not agree	7228	90.3 (89.4, 91.3)	550	64.8 (59.5, 70.1)	250	51.2 (44.2, 58.2)
Somewhat agree	555	7.3 (6.5, 8.1)	173	25.4 (20.5, 30.2)	107	25.6 (19.3, 31.8)
Strongly/very strongly agree	151	2.3 (1.8, 2.8)	55	9.9 (6.1, 13.6)	87	23.2 (17.5, 28.9)
Adjusted prevalence difference (95% CI; q-value)^§^	Referent		6.8 (3.1, 10.4; < 0.001)		19.6 (13.8, 25.5; < 0.001)	

Query and response	Anticipated future firearm use when political violence is justified^‡^					
	I will shoot someone with a gun					
	Not likely		Somewhat likely		Very/extremely likely	
	Unweighted n	Weighted % (95% CI)	Unweighted n	Weighted % (95% CI)	Unweighted n	Weighted % (95% CI)
Which of the following comes closer to your view of what a second civil war might look like?*						
Like an insurgency or guerrilla war, with small groups attacking specific targets or people	7746	89.2 (88.2, 90.3)	261	70.3 (62.4, 78.3)	107	71.1 (59.8, 82.4)
Like the first Civil War in the United States, with opposing armies and large battles	658	10.8 (9.7, 11.8)	57	29.7 (21.7, 37.6)	34	28.9 (17.6, 40.2)
Adjusted prevalence difference (95% CI; q-value)^†^	Referent		14.3 (6.4, 22.1; < 0.001)		11.4 (0.1, 22.6; 0.05)	
*How much do you agree or disagree with each of the following statements?*^*†*^						
In the next few years, there will be civil war in the United States						
Do not agree	5972	67.3 (65.9, 68.6)	126	32.9 (25.4, 40.5)	31	18.8 (9.8, 27.7)
Somewhat agree	2335	28.1 (26.8, 29.4)	152	48.2 (40.0, 56.5)	61	37.9 (26.9, 48.9)
Strongly/very strongly agree	374	4.6 (4.0, 5.3)	48	18.8 (12.1, 25.6)	53	43.3 (31.6, 55.1)
Adjusted prevalence difference (95% CI; q-value)^§^	Referent		12.6 (5.9, 19.3; < 0.001)		37.0 (25.5, 48.5; < 0.001)	
*The United States needs a civil war to set things right*						
Do not agree	7784	89.2 (88.2, 90.1)	202	49.0 (40.9, 57.2)	55	30.4 (20.4, 40.4)
Somewhat agree	713	8.3 (7.5, 9.1)	88	34.2 (26.0, 42.4)	35	24.9 (14.6, 35.2)
Strongly/very strongly agree	201	2.6 (2.1, 3.0)	39	16.8 (9.9, 23.6)	55	44.7 (33.0, 56.4)
Adjusted prevalence difference (95% CI; q-value)^§^	Referent		12.9 (6.6, 19.3; < 0.001)		40.6 (29.1, 52.1; < 0.001)	

*Some people talk about a second civil war in the United States. Which of the following comes closer to your view of what a second civil war might look like? 1. A second civil war would be like the first Civil War in the United States, with opposing armies and large battles. 2. A second civil war would be like an insurgency or guerrilla war, with small groups attacking specific targets or people

^†^Adjusted prevalence difference is for the “like the first Civil War” response

^‡^Thinking now about the future and all the changes it might bring, how likely is it that you will use a gun in any of the following ways in the next few years—in a situation where you think force or violence is justified to advance an important political objective?

^§^Adjusted prevalence difference is for the strongly/very strongly agree response

Adjusted models include age, race and ethnicity, gender, education, income, Census division, and rurality. Adjusted differences are for the strongly/very strongly agree comparison. Q-values represent the probability that the given difference would be a false discovery; they represent the expected proportion of “false positives” that would be seen among the collection of all differences whose q-values were at or below the given q-value

## Discussion

In these data from 2023’s Wave 2 of our nationally representative longitudinal survey, the expectation that civil war was coming and the belief that it was necessary were both uncommon. The expectation had become significantly less common from 2022 to 2023 (Wintemute et al. 2024a), with the prevalence of strong or very strong agreement with the assertion that “in the next few years, there will be civil war in the United States” decreasing by more than half (from 13.7 to 5.7%). These are hopeful findings, reinforced by the fact that in both 2022 and 2023, large majorities of respondents reported that political violence was never justified, and of the minorities who did consider violence justified to advance political objectives, large majorities said that they were unwilling to participate in such violence themselves (Wintemute et al. 2023a, 2024a).

But 2022 was a federal election year, and 2023 was not. In 2024, a presidential election year characterized by increasing political animosity and by violent rhetoric from some leading political figures (Blake 2024), expectations of and support for civil war may well increase (Armed Conflict Location Event Data Project (ACLED) 2024). Already in 2023, according to our findings, nearly 40% of those who believed most strongly that civil war was coming also believed that it was needed.

It is a particularly concerning finding of this 2023 survey that expectations of and a perceived need for civil war are higher among subsets of the population that are also more likely than others to view political violence as justified and frequently more willing than others to engage in such violence themselves (Wintemute et al. 2023a, 2024b, 2024c, 2023b, 2022b). The long list of these subsets includes Republicans, MAGA Republicans, extreme conservatives, persons in strong or moderate agreement with racist beliefs or statements of the potential need for violence to effect social change, persons who strongly approve of specified extreme right-wing political organizations and movements, owners of assault-type rifles, firearm owners who purchased firearms in 2020 or later, and firearm owners who carry firearms in public all or nearly all the time.

Only minorities of respondents expected formal conflict (“opposing armies and large battles”), even in the subgroups where expectation of and perceived need for civil war were highest. There is consensus among scholars as well that while formal conflict is extremely unlikely, sporadic outbreaks of large-scale political violence, targeted attacks intended to disrupt the electoral process, and insurgency remain real possibilities (Walter 2022; Simon and Stevenson 2023; Armed Conflict Location Event Data Project (ACLED) 2024; Kleinfeld 2022; Federal Bureau of Investigation and Department of Homeland Security 2023).

What should be the response to these findings? First, the large majority of the population who reject political violence should make their opposition clear; a climate of non-support for political violence may reduce the likelihood that it will occur. We base that assertion on the premise that, if violence is a health problem, then participation in violence is a health behavior. Such behaviors can be influenced by family members (Michaelson et al. 2021), friends (Houle et al. 2017), coworkers (Pruckner et al. 2020), social media contacts (Kanchan and Gaidhane 2023), and well-known public figures (Hoffman et al. 2017).

Second, violence being a health problem, public health, public safety, healthcare system, and clinical health professionals need to collaborate in preparing for and preventing outbreaks of political violence of a scale that could exceed the capabilities of many healthcare delivery systems.

More broadly, structural reform and behavior change may matter most; intervening on underlying attitudes and beliefs has disappointingly little effect (Kleinfeld 2023). Thoughtful recommendations for action on policy and social change have been developed (Tisler and Norden 2024; Clapman 2024; Carey et al. 2023; Morales-Doyle et al. 2023). Specific recommendations for preventing electoral violence have also been proposed (Ware 2024). To all these should be added this recommendation for action by individual members of the public: “if you see something, say something” (Department of Homeland Security 2024). Many prevention measures depend on critical information about threatened violence getting to those in a position to intervene against the threat (National Counterterrorism Center 2021).

### Limitations

Several technical limitations exist. The findings are cross-sectional and subject to sampling error and nonresponse bias. Respondents and nonrespondents differed in age and gender, which are related to support for political violence. Arguably, nonresponse was most important in Wave 1; the 84% response rate for Wave 2 was high. Some outcomes are uncommon, with response counts < 100. The large study sample notwithstanding, the estimates remain vulnerable to bias from sources such as inattentive or strategic responses.

External events (or their absence) may have affected our findings. The survey closed just before the federal criminal indictment of Donald Trump was handed down; support for violence to return him to the White House increased immediately thereafter (Pape 2023); expectations of and support for civil war might have as well. Given the nature of the topic, strategic responding is a consideration.

## Conclusion

Findings from this large, nationally representative longitudinal survey indicate that while expectations of and support for civil war are uncommon, they are higher among many subsets of the population that are at greater risk for committing political violence. These findings can help guide prevention efforts, which are urgently needed.

### Supplementary Information

**Additional file 1**. Questions that supplied data for this analysis, additional methods and results text, and 13 tables

## Data Availability

No datasets were generated or analysed during the current study.

## Abbreviations

SD: Standard deviation
CI: Confidence interval
