# Supplementary material for: Expectations of and perceived need for civil war in the USA: findings from a 2023 nationally representative survey
Source: Inj Epidemiol. 2024 Aug 29;11:40. doi: 10.1186/s40621-024-00521-5 (PMC11360528; doi:10.1186/s40621-024-00521-5)
Supplement: Supplementary file 1 — Additional file 1. Questions that supplied data for this analysis, additional methods and results text, and 13 tables [file 40621_2024_521_MOESM1_ESM.pdf]

Supplement

Expectations of and Perceived Need for Civil War in the USA:

Findings from a 2023 Nationally Representative Survey

This supplement has been provided by the authors to give readers additional information about the work.

Page	Title
2	Questions that supplied data for this study
9	Additional methods text
12	Additional results text
14	References
15	Table S1. Sociodemographic characteristics of respondents
18	Table S2. Sociodemographic characteristics (unweighted) of respondents and non-respondents in the 2022 and 2023 political violence surveys
20	Table S3. Association between political party affiliation and expectations and perceived need for civil war in the United States
22	Table S4. Association between self-reported MAGA status and expectations and perceived need for civil war in the United States
23	Table S5. Association between political ideology and expectations and perceived need for civil war in the United States
25	Table S6. Association between racist beliefs and expectations and perceived need for civil war in the United States
26	Table S7. Association between pro-violence beliefs and expectations and perceived need for civil war in the United States
27	Table S8. Association between approval of extreme right-wing organizations and movements and expectations and perceived need for civil war in the United States
28	Table S9. Association between approval of specific extreme right-wing organizations and movements and expectations and perceived need for civil war in the United States
36	Table S10. Association between firearm ownership status and expectations and perceived need for civil war in the United States
37	Table S11. Association between types(s) of firearm owned and expectations and perceived need for civil war in the United States
38	Table S12. Association between recency of firearm purchase and expectations and perceived need for civil war in the United States
40	Table S13. Association between frequency of firearm carrying and expectations and perceived need for civil war in the United States

QUESTIONS THAT SUPPLIED DATA FOR THIS STUDY

Response options are presented here in order from negative to positive (e.g., “not important” to “extremely important”). Respondents were randomized 1:1 to receive responses in that order or the reverse.

In the list below, questions or items that were repeated or adapted from prior surveys contain citations to those surveys.

Domain 1: civil war

Q: Some people talk about a second civil war in the United States. Which of the following comes closer to your view of what a second civil war might look like?

> A second civil war would be like the first Civil War in the United States, with opposing armies and large battles.
>
> OR
>
> A second civil war would be like an insurgency or guerrilla war, with small groups attacking specific targets or people.

Q: How much do you agree or disagree with each of the following statements?

a. In the next few years, there will be civil war in the United States.[1]

b. The United States needs a civil war to set things right.

1. Do not agree
2. Somewhat agree
3. Strongly agree
4. Very strongly agree

Domain 2: race and ethnicity

Q: How much do you agree or disagree with each of the following statements about people in America today?

a. White people benefit from advantages in society that Black people do not have.[2] (Reverse coded)

b. Discrimination against whites is as big a problem as discrimination against Blacks and other minorities.[3]

…

d. In America, native-born white people are being replaced by immigrants.

e. Having more Black Americans, Latinos, and Asian Americans is good for the country.[4] (Reverse coded)

1. Do not agree
2. Somewhat agree
3. Strongly agree
4. Very strongly agree

Domain 3: violence to effect social change

Q: How much do you agree or disagree with the following statements about democracy in the United States?

d. If elected leaders will not protect American democracy, the people must do it themselves, even if it requires taking violent actions.[2]

Q: How much do you agree or disagree with each of the following statements about people in America today?

c. Our American way of life is disappearing so fast that we may have to use force to save it.[2]

1. Do not agree
2. Somewhat agree
3. Strongly agree
4. Very strongly agree

Q: People have many different views about American society. How much do you agree or disagree with each of the following?

c. Because things have gotten so far off track, true American patriots may have to resort to violence in order to save our country.[5]

1. Do not agree
2. Somewhat agree
3. Strongly agree

4. Very strongly agree

Domain 4: Party affiliation and political ideology

Q: Generally speaking, do you think of yourself as...*Select one answer only.*

1. Republican

2. Democrat

3. Independent (A)

6. Something else (A)

(Asked if Republican)

Q: Would you call yourself a...*Select one answer only.*

1. Strong Republican

2. Not very strong Republican

(Asked if Democrat)

Q: Would you call yourself a...*Select one answer only.*

1. Strong Democrat

2. Not very strong Democrat

(Asked if Independent or Something else)

Q: Do you think of yourself as closer to the...*Select one answer only.*

1. Republican Party

2. Democratic Party

3. Do not lean either way

(Asked if Republicans OR Leans Republican)

Q: Do you think of yourself as a MAGA Republican?

1. No

2. Yes

(Asked if Not MAGA Republican OR Democrat / Leans Democrat)

Q: Do you think of yourself as a supporter of the MAGA movement?

1. No

2. Yes

Domain 5: Firearm ownership

Ownership Status

Q: Do you happen to keep any guns in your home or garage?

1. Yes

2. No

(Asked if the response to the prior question was "yes.")

Q: Do any of these guns personally belong to you?

1. Yes

2. No

Type(s) of Firearm Owned

(Asked if the response to the prior question was "yes." Presented with firearm types in rows and responses in columns. Respondents gave separate answers for each firearm type.)

Q: Do you personally own any of the following types of guns?

a. Handguns

b. Rifles

c. Shotguns

d. Other types of guns

1. Yes

2. No

(Asked if the response to "rifles" in the prior question was "yes.")

Q: Do you own any rifles of the type sometimes called tactical rifles, or modern sporting rifles, or assault rifles, such as an AR-15, an AK-47, or an SKS?

1. Yes

2. No

Recency of Purchase

(Asked if the response to the personal ownership question was "yes.")

Q: Did you buy any guns this year, in 2022?

1. Yes

2. No

Q: Did you buy any guns in 2021?

1. Yes

2. No

Q: Did you buy any guns in 2020?

1. Yes

2. No

Q: When you bought guns in (insert earliest year from the series above) did you already own any guns?

1. Yes

2. No

Carrying Behavior

(Asked of all respondents)

Q: In the last year, have you carried a loaded gun (handgun, rifle, or shotgun) on your person when you were out in public? Do not include hunting, time at a shooting range, or similar activities.

1. Yes

2. No

(Asked if the response to the prior question was "yes.")

Q: In the last year, and on days when you were out in public, how often have you carried a loaded gun on your person? Again, do not include hunting, time at a shooting range, or similar activities.

1. Not often at all

2. Less than half the time

3. About half the time

4. More than half the time

5. All (or nearly all) the time

Domain 6: Approval of specified organizations and movements

Q: How much do you approve or disapprove of these named groups and organizations?

Proud Boys

Oath Keepers

Three Percenters

QAnon

Q: How much do you approve or disapprove of these political or social movements?

The militia movement

The white supremacy movement

The Christian nationalist movement

The boogaloo movement

1. Do not approve
2. Somewhat approve
3. Strongly approve
4. Very strongly approve
5. I don’t know enough about this group or organization to rate it [ANCHOR]
6. I have never heard of this group or organization [ANCHOR]

Question from the KnowledgePanel profile questionnaire that supplied data for this study

Political ideology

Q: In general, do you think of yourself as...

1. Extremely liberal
2. Liberal
3. Slightly liberal
4. Moderate/middle of the road
5. Slightly conservative
6. Conservative
7. Extremely conservative

ADDITIONAL METHODS TEXT

Construction of Measures

Party affiliation

Party affiliation was derived following a procedure used since the 1950s by the American National Election Studies.[6] As described in the report of a previous KnowledgePanel survey,

> Respondents were first asked, "Generally speaking, do you think of yourself as" (response choices: "Republican," "Democrat," "Independent," "Another party," "No preference"). Republicans/Democrats were then asked, "Would you call yourself a" (response choices: "Strong Republican/Democrat," "Not very strong Republican/Democrat"). All others were asked the follow-up question: "Do you think of yourself as closer to the" (response choices: "Republican Party," "Democratic Party").[7]

Respondents who answered that follow-up question were coded by Ipsos as "Leans Republican/Democrat." Respondents who refused to answer (n=309, 3.6% of the sample) were coded by Ipsos as "undecided/independent/other."

MAGA status

Respondents who identified as Republican, or as independent or something else but closer to the Republican Party than to the Democratic Party, were asked, "Do you think of yourself as a

MAGA Republican?" All who responded "no" to that question and all other respondents were asked, "Do you think of yourself as a supporter of the MAGA movement?"

Political ideology

Political ideology was reported by panel members in responding to the question, "In general, do you think of yourself as…" with 7 response options ranging from "extremely liberal" to "extremely conservative" and with "moderate/middle of the road" as a midpoint.

Racist beliefs, beliefs regarding violence to effect social change, affiliation with organizations and movements[8]

Individual item responses these exposures were coded ordinally (e.g., do not agree= 0, somewhat agree= 1, strongly agree= 2, very strongly agree=3) and summed for each respondent for each of the 3 exposures. Summed scores were normalized to a range from 0 to 1, with 0 and 1 representing the minimum and maximum theoretically possible scores, respectively. Respondents' normalized scores were then categorized according to their position on that range (e.g., strong agreement, normalized score> 0.66… and ≤1; moderate agreement, normalized score> 0.33… and ≤0.66; weak agreement, normalized score> 0 and ≤0.33…; non-agreement, normalized score= 0). Modified procedures allowed for inclusion of respondents with missing values for up to half the items for any exposure (Table 1 and Supplement).

For each exposure, we calculated Cronbach's α using individual item scores to assess the internal consistency of those items, with bootstrapped 95% confidence intervals (CI) based on 500 samples.

Firearm ownership and use[9]

Respondents were categorized as personal firearm owners, nonowners with firearms at home, and nonowners without firearms at home. Firearm owners were categorized in 4 groups based on the type(s) of firearm they owned: assault-type rifle owners (owns 1 or more assault-type rifles; may own firearms of other types), other rifle owners (owns 1 or more rifles that are not-assault type rifles; does not own assault-type rifles; may own firearms of other types), handgun-only owners (owns 1 or more handguns and no firearms of other types), other owners (owns any combination of handguns, shotguns, and firearms of other types; does not own rifles). Firearm owners were also categorized in 2 groups by recency of most recent purchase: 2020 or later, and 2019 or earlier. Finally, owners were categorized in 4 groups based on their frequency of carrying a loaded firearm on their person when out in public (with sporting use excluded): never/not often at all; less than half the time/about half the time/more than half the time; and always/nearly always.

Statistical analysis

We examined the following models for adjusting prevalence differences:

Model 0: unadjusted;

Model 1: adjusted for age (numerical), race and ethnicity (White, Non-Hispanic; Black, Non-Hispanic; Other, Non-Hispanic; Hispanic; 2+ Races, Non-Hispanic), and gender (Male, Female);

Model 2: additionally adjusted for income (Less than $10,000, $10,000 to $24,999, $25,000 to $49,999, $50,000 to $74,999, $75,000 to $99,999, $100,000 to $149,999, $150,000 or more),

education (No high school diploma or GED, High school graduate (high school diploma or the equivalent GED), Some college or Associate's degree, Bachelor's degree, Master’s degree or higher), and Census division (New England, Mid-Atlantic, East-North Central, West-North Central, South Atlantic, East-South Central, West-South Central, Mountain, Pacific);

Model 3: additionally adjusted for rurality (Urban, Rural; derived from Rural-Urban Commuting Codes matched to census tracts (https://www.ers.usda.gov/data-products/rural-urban-commuting-area-codes/).

Model 3 was selected; findings from Model 3 appear in the ‘Adjusted prevalence difference” rows in tables. Q-values were also produced using Model 3.

ADDITIONAL RESULTS TEXT

Four items in the 2023 survey had nonresponse percentages above 2.0%:

Q: People have many different views about society in the United States. How much do you agree or disagree with each of the following?

There is a storm coming soon that will sweep away the elites in power and restore the rightful leaders.

Nonresponse= 2.2%.

Q: How much do you agree or disagree with each of the following statements about people in the United States today?

A group of people in this country is trying to replace native-born Americans with immigrants and people of color who share their political views.

Nonresponse= 2.2%.

Having more Black Americans, Latinos, and Asian Americans is good for the country.

Nonresponse= 3.1%.

Q: Some people talk about a second civil war in the United States. Which of the following comes closer to your view of what a second civil war might look like?

A second civil war would be like the first Civil War in the United States, with opposing armies and large battles.

OR

A second civil war would be like an insurgency or guerrilla war, with small groups attacking specific targets or people.

Nonresponse= 5.7%

REFERENCES

1. Zogby. Will the US have another civil war? 2021 Feb 4. https://zogbyanalytics.com/news/997-the-zogby-poll-will-the-us-have-another-civil-war.

2. Pew Research Center. Deep divisions in Americans' views of nation's racial history – and how to address it. 2021 August. https://www.pewresearch.org/politics/2021/08/12/deep-divisions-in-americans-views-of-nations-racial-history-and-how-to-address-it/.

3. Cox D, Lienesch R, Jones RP. Beyond economics: fears of cultural displacement pushed the white working class to Trump | PRRI/The Atlantic Report. Public Religion Research Institute. 2019 May 17. https://www.prri.org/research/white-working-class-attitudes-economy-trade-immigration-election-donald-trump/.

4. Pew Research Center. Americans see advantages and challenges in country's growing racial and ethnic diversity. 2019 May. https://www.pewresearch.org/social-trends/2019/05/08/americans-see-advantages-and-challenges-in-countrys-growing-racial-and-ethnic-diversity/.

5. IFYC – PRRI Survey on Religion & COVID-19 Vaccine Trust. 2021 March. https://www.prri.org/wp-content/uploads/2021/05/Topline-IFYC-PRRI-Survey-on-Religion-and-COVID-19-Vaccine-Trust-v2_final.pdf.

6. American National Election Studies. https://electionstudies.org/.

7. Malhotra N, Kuo AG. Emotions as moderators of information cue use citizen attitudes toward Hurricane Katrina. Am Politics Res. 2009;37:301-326.

8. Wintemute GJ, Velasquez B, Li Y, Tomsich EA, Reeping PM, Robinson SL. Racist and pro-violence beliefs, approval of extreme right-wing political organizations and movements, and support for political violence in the United States. [Preprint.] *SocArXiv*. 2023. Online publication Dec 4. Available at: https://osf.io/preprints/socarxiv/c9vtr.

9. Wintemute GJ, Crawford A, Robinson S, Tomsich E, Reeping P, Schleimer J, Pear V. Firearm ownership and support for political violence in the United States. JAMA Netw Open. 2024;7(4):e243623.

Table S1. Sociodemographic characteristics of respondents

Characteristic	2023 Respondents* (n= 9385)	
	Unweighted n	Weighted % (95% CI)
**Age**		
18-24	310	10.3 (9.2, 11.5)
25-34	856	16.8 (15.6, 18.0)
35-44	1252	18.5 (17.4, 19.6)
45-54	1255	14.3 (13.4, 15.2)
55-64	2043	17.6 (16.7, 18.5)
65-74	2342	14.5 (13.8, 15.3)
75+	1327	8.0 (7.4, 8.5)
Non-response	0	0.0 (0.0, 0.0)
**Gender**		
Female	3866	50.7 (49.4, 52.1)
Male	5340	47.0 (45.7, 48.4)
Transgender	45	0.5 (0.3, 0.7)
Non-binary	59	0.8 (0.5, 1.0)
Other	21	0.3 (0.1, 0.5)
Non-response	54	0.7 (0.4, 0.9)
**Race/Ethnicity**		
White, Non-Hispanic	7014	62.7 (61.2, 64.1)
Black, Non-Hispanic	748	12.0 (10.9, 13.0)
Hispanic, any race	1016	16.9 (15.7, 18.1)
American Indian or Alaska Native, Non-Hispanic	47	1.1 (0.7, 1.5)
Asian American or Pacific Islander, non-Hispanic	277	5.5 (4.7, 6.2)
Some other race, Non-Hispanic	19	0.1 (0.1, 0.2)
2+ Races, Non-Hispanic	264	1.8 (1.4, 2.2)
Non-response	0	0.0 (0.0, 0.0)
**Marital status**		
Now married	5961	56.2 (54.8, 57.6)
Widowed	582	3.9 (3.5, 4.4)
Divorced	1010	8.2 (7.6, 8.8)
Separated	122	1.4 (1.1, 1.8)
Never married	1710	30.2 (28.8, 31.6)
Non-response	0	0.0 (0.0, 0.0)

Table S1, continued.

Characteristic	2023 Respondents* (n= 9385)	
	Unweighted n	Weighted % (95% CI)
**Education**		
No high school diploma or GED	416	9.5 (8.4, 10.5)
High school graduate (diploma, GED)	2002	28.2 (26.9, 29.6)
Some college or Associate's degree	2773	27.1 (25.9, 28.3)
Bachelor's degree	2337	20.1 (19.1, 21.1)
Master's degree or higher	1857	15.1 (14.2, 15.9)
Non-response	0	0.0 (0.0, 0.0)
**Household Income**		
Less than $10,000	233	3.9 (3.2, 4.5)
$10,000 to $24,999	727	8.9 (8.1, 9.8)
$25,000 to $49,999	1617	17.0 (15.9, 18.0)
$50,000 to $74,999	1631	16.3 (15.3, 17.4)
$75,000 to $99,999	1499	13.2 (12.3, 14.1)
$100,000 to $149,999	1734	17.9 (16.8, 18.9)
$150,000 or more	1944	22.8 (21.6, 23.9)
Non-response	0	0.0 (0.0, 0.0)
**Employment**		
Working - as a paid employee	4291	52.9 (51.6, 54.3)
Working - self-employed	709	7.2 (6.5, 8.0)
Not working - on temporary layoff from a job	35	0.5 (0.3, 0.7)
Not working - looking for work	272	5.2 (4.4, 5.9)
Not working - retired	3367	21.3 (20.4, 22.2)
Not working - disabled	286	4.5 (3.9, 5.2)
Not working - other	425	8.3 (7.4, 9.2)
Non-response	0	0.0 (0.0, 0.0)
**Census division**		
New England	374	4.7 (4.1, 5.3)
Mid-Atlantic	1001	12.6 (11.6, 13.5)
East-North Central	1370	14.3 (13.3, 15.2)
West-North Central	676	6.4 (5.8, 7.0)
South Atlantic	1881	20.5 (19.4, 21.6)
East-South Central	538	5.8 (5.1, 6.5)
West-South Central	965	11.9 (10.9, 12.8)
Mountain	825	7.6 (6.9, 8.3)
Pacific	1755	16.3 (15.3, 17.3)
Non-response	0	0.0 (0.0, 0.0)

This table previously appeared as part of Table 1 in Wintemute GJ, Robinson SL, Crawford A, Tomsich EA, Reeping PM, Shev AB, Velasquez B, Tancredi D. Single-year change in views of democracy and society and support for political violence in the USA: findings from a 2023 nationally representative survey. Inj Epidemiol. 2024 May 21;11:20.

Table S2. Sociodemographic characteristics (unweighted) of respondents and non-respondents in the 2022 and 2023 political violence surveys

Characteristic	2022 (Wave1)				Wave1 respondents who left the panel prior to wave2 (n = 1,807)		2023 (Wave2)			
	Respondents (n = 12,947)		Non-respondents (n = 8,318)				Respondents (n = 9,385)		Non-respondents (n = 1,755)	
	Unweighted n	Unweighted %	Unweighted n	Unweighted %	Unweighted n	Unweighted %	Unweighted n	Unweighted %	Unweighted n	Unweighted %
Age										
18-24	488	3.8	1059	12.7	86	4.8	310	3.3	92	5.2
25-34	1309	10.1	1411	17.0	210	11.6	856	9.1	243	13.8
35-44	1884	14.6	1732	20.8	326	18.0	1252	13.3	306	17.4
45-54	1847	14.3	1599	19.2	335	18.5	1255	13.4	257	14.6
55-64	2794	21.6	1254	15.1	391	21.6	2043	21.8	360	20.5
65-74	2952	22.8	861	10.4	313	17.3	2342	25.0	297	16.9
75+	1673	12.9	402	4.8	146	8.1	1327	14.1	200	11.4
Non-response	0	0.0	0	0.0	0	0.0	0	0.0	0	0.0
Gender										
Male	7158	55.3	3993	48.0	854	47.3	5437	57.9	867	49.4
Female	5789	44.7	4325	52.0	953	52.7	3948	42.1	888	50.6
Non-response	0	0.0	0	0.0	0	0.0	0	0.0	0	0.0
Race and ethnicity										
Black, non-Hispanic	1097	8.5	1039	12.5	170	9.4	749	8.0	178	10.1
Hispanic	1504	11.6	1561	18.8	237	13.1	1016	10.8	251	14.3
White, non-Hispanic	9493	73.3	5030	60.5	1272	70.4	7014	74.7	1207	68.8
Other, non-Hispanic	499	3.9	370	4.4	77	4.3	346	3.7	76	4.3
2+ races, non-Hispanic	354	2.7	318	3.8	51	2.8	260	2.8	43	2.5
Non-response	0	0.0	0	0.0	0	0.0	0	0.0	0	0.0
Marital status										
Now married	8074	62.4	4460	53.6	1089	60.3	5961	63.5	1024	58.3
Widowed	770	5.9	303	3.6	82	4.5	582	6.2	106	6.0
Divorced	1456	11.2	858	10.3	240	13.3	1010	10.8	206	11.7
Separated	193	1.5	191	2.3	34	1.9	122	1.3	37	2.1
Never married	2454	19.0	2506	30.1	362	20.0	1710	18.2	382	21.8
Non-response	0	0.0	0	0.0	0	0.0	0	0.0	0	0.0
Education										
No high school diploma or GED	624	4.8	694	8.3	121	6.7	416	4.4	87	5.0
High school graduate or GED	2813	21.7	2084	25.1	452	25.0	2002	21.3	359	20.5
Some college or Associate degree	3896	30.1	2649	31.8	584	32.3	2773	29.5	539	30.7
Bachelor's degree	3133	24.2	1726	20.8	372	20.6	2337	24.9	424	24.2
Master's degree or higher	2481	19.2	1165	14.0	278	15.4	1857	19.8	346	19.7
Non-response	0	0.0	0	0.0	0	0.0	0	0.0	0	0.0

Table S2, continued

Characteristic	2022 (Wave1)				Wave1 respondents who left the panel prior to wave2 (n = 1,807)		2023 (Wave2)			
	Respondents (n = 12,947)		Non-respondents (n = 8,318)				Respondents (n = 9,385)		Non-respondents (n = 1,755)	
	Unweighted n	Unweighted %	Unweighted n	Unweighted %	Unweighted n	Unweighted %	Unweighted n	Unweighted %	Unweighted n	Unweighted %
Household Income										
< $10,000	371	2.9	410	4.9	72	4.0	233	2.5	66	3.8
$10,000 - $24,999	1078	8.3	793	9.5	189	10.5	727	7.7	162	9.2
$25,000 - $49,999	2232	17.2	1558	18.7	318	17.6	1617	17.2	297	16.9
$50,000 - $74,999	2236	17.3	1427	17.2	313	17.3	1631	17.4	292	16.6
$75,000 - $99,999	1999	15.4	1203	14.5	236	13.1	1499	16.0	264	15.0
$100,000 - $149,999	2410	18.6	1461	17.6	336	18.6	1734	18.5	340	19.4
≥ $150,000	2621	20.2	1466	17.6	343	19.0	1944	20.7	334	19.0
Non-response	0	0.0	0	0.0	0	0.0	0	0.0	0	0.0
Employment										
Working full-time	5645	43.6	4514	54.3	889	49.2	3869	41.2	887	50.5
Working part-time	1620	12.5	1342	16.1	258	14.3	1133	12.1	229	13.0
Not working	5682	43.9	2462	29.6	660	36.5	4383	46.7	639	36.4
Non-response	0	0.0	0	0.0	0	0.0	0	0.0	0	0.0
Census division										
New England	509	3.9	297	3.6	73	4.0	374	4.0	62	3.5
Mid-Atlantic	1407	10.9	915	11.0	191	10.6	1001	10.7	215	12.3
East-North Central	1878	14.5	1117	13.4	262	14.5	1370	14.6	246	14.0
West-North Central	952	7.4	597	7.2	137	7.6	676	7.2	139	7.9
South Atlantic	2538	19.6	1652	19.9	326	18.0	1881	20.0	331	18.9
East-South Central	737	5.7	579	7.0	117	6.5	538	5.7	82	4.7
West-South Central	1371	10.6	1093	13.1	207	11.5	965	10.3	199	11.3
Mountain	1125	8.7	573	6.9	156	8.6	825	8.8	144	8.2
Pacific	2430	18.8	1495	18.0	338	18.7	1755	18.7	337	19.2
Non-response	0	0.0	0	0.0	0	0.0	0	0.0	0	0.0

Mean (SD) ages were as follows: Wave 1 responders, 55.7 (16.7); Wave 1 non-responders, 45.4 (16.8); Wave 1 respondents who left the panel prior to Wave 2, 52.17 (16.2); Wave 2 responders, 56.99 (16.5); Wave 2 non-responders, 52.47 (17.5).

This table previously appeared as Table S1 in the supplement to Wintemute GJ, Robinson SL, Crawford A, Tomsich EA, Reeping PM, Shev AB, Velasquez B, Tancredi D. Single-year change in views of democracy and society and support for political violence in the USA: findings from a 2023 nationally representative survey. Inj Epidemiol. 2024 May 21;11:20.

Table S3. Association between political party affiliation and expectations and perceived need for civil war in the United States

Query and Response	Party Affiliation							
	Strong Democrat		Not Very Strong Democrat		Leans Democrat		Independent/Other	
	Unweighted n	Weighted % (95% CI)	Unweighted n	Weighted % (95% CI)	Unweighted n	Weighted % (95% CI)	Unweighted n	Weighted % (95% CI)
Which of the following comes closer to your view of what a second civil war might look like?*								
Like an insurgency or guerrilla war, with small groups attacking specific targets or people.	1539	88.4 (85.9, 90.9)	982	89.0 (86.4, 91.5)	1078	94.1 (92.1, 96.2)	1172	84.8 (81.9, 87.8)
Like the first Civil War in the United States, with opposing armies and large battles.	113	11.6 (9.1, 14.1)	99	11.0 (8.5, 13.6)	42	5.9 (3.8, 7.9)	136	15.2 (12.2, 18.1)
Adjusted prevalence difference (95% CI; q-value)[†]	-1.2 (-5.0, 2.6; 0.75)		-2.9 (-6.7, 0.9; 0.44)		-4.4 (-7.9, -0.9; 0.07)		Referent	
How much do you agree or disagree with each of the following statements?								
In the next few years, there will be civil war in the United States.								
Do not agree	1189	69.2 (66.3, 72.2)	773	68.7 (65.0, 72.5)	815	69.0 (65.3, 72.6)	887	60.9 (57.3, 64.5)
Somewhat agree	428	26.2 (23.4, 29.0)	281	26.2 (22.7, 29.7)	285	26.0 (22.6, 29.4)	408	32.6 (29.1, 36.0)
Strongly/very strongly agree	62	4.6 (3.1, 6.0)	47	5.0 (3.2, 6.8)	40	5.0 (2.9, 7.2)	76	6.5 (4.6, 8.4)
Adjusted prevalence difference (95% CI; q-value)[§]	-1.1 (-3.5, 1.3; 0.57)		-1.2 (-3.9, 1.5; 0.57)		0.3 (-2.6, 3.2; 0.84)		Referent	
The United States needs a civil war to set things right.								
Do not agree	1588	91.1 (89, 93.3)	1040	92.7 (90.3, 95.0)	1092	93.0 (90.8, 95.3)	1176	82.1 (79.1, 85.0)
Somewhat agree	70	6.3 (4.5, 8.2)	45	4.6 (2.8, 6.4)	35	4.6 (2.8, 6.4)	143	13.1 (10.3, 15.8)
Strongly/very strongly agree	25	2.5 (1.4, 3.7)	19	2.7 (1.1, 4.3)	14	2.4 (0.8, 3.9)	60	4.9 (3.3, 6.4)
Adjusted prevalence difference (95% CI; q-value)[§]	-1.3 (-3.2, 0.7; 0.45)		-1.6 (-3.8, 0.6; 0.44)		-1.2 (-3.4, 1.0; 0.56)		Referent	

Table S3, continued.

Query and Response	Party Affiliation					
	Leans Republican		Not Very Strong Republican		Strong Republican	
	Unweighted n	Weighted % (95% CI)	Unweighted n	Weighted % (95% CI)	Unweighted n	Weighted % (95% CI)
Which of the following comes closer to your view of what a second civil war might look like?*						
Like an insurgency or guerrilla war, with small groups attacking specific targets or people.	991	90.4 (87.5, 93.3)	967	88.8 (85.9, 91.7)	1435	84.1 (81.3, 86.8)
Like the first Civil War in the United States, with opposing armies and large battles.	72	9.6 (6.7, 12.5)	96	11.2 (8.3, 14.1)	194	15.9 (13.2, 18.7)
Adjusted prevalence difference (95% CI; q-value)†	-0.5 (-4.4, 3.4; 0.84)		0.9 (-3.1, 5.0; 0.75)		6.1 (2.2, 10.1; 0.01)	
How much do you agree or disagree with each of the following statements?						
In the next few years, there will be civil war in the United States.						
Do not agree	724	62.6 (58.5, 66.6)	771	67.9 (64.1, 71.6)	1000	56.5 (53.3, 59.7)
Somewhat agree	317	31.2 (27.3, 35.2)	291	28.9 (25.3, 32.6)	564	33.0 (30.0, 36.0)
Strongly/very strongly agree	67	6.2 (4.2, 8.1)	40	3.2 (1.8, 4.7)	148	10.5 (8.3, 12.7)
Adjusted prevalence difference (95% CI; q-value)§	1.9 (-0.9, 4.6; 0.45)		-1.2 (-3.6, 1.3; 0.57)		6.0 (3.0, 8.9; 0.001)	
The United States needs a civil war to set things right.						
Do not agree	921	82.4 (79.2, 85.6)	961	86.3 (83.4, 89.2)	1308	76.5 (73.8, 79.3)
Somewhat agree	155	13.5 (10.7, 16.3)	110	11.0 (8.5, 13.6)	293	15.8 (13.6, 18.0)
Strongly/very strongly agree	38	4.1 (2.2, 5.9)	30	2.7 (1.1, 4.3)	110	7.6 (5.7, 9.5)
Adjusted prevalence difference (95% CI; q-value)§	0.5 (-1.9, 3.0; 0.75)		-0.6 (-2.8, 1.6; 0.75)		4.4 (1.8, 6.9; 0.006)	

Details on assignment of party affiliation are in the Supplemental Methods Text section of this supplement (p 11).

*Q: Some people talk about a second civil war in the United States. Which of the following comes closer to your view of what a second civil war might look like? R1: A second civil war would be like the first Civil War in the United States, with opposing armies and large battles. R2: A second civil war would be like an insurgency or guerrilla war, with small groups attacking specific targets or people.

† Response options were do not agree, somewhat agree, strongly agree, very strongly agree. Findings are combined for the strongly/very strongly agree responses.

§ Adjusted models include age, race and ethnicity, gender, education, income, Census division, and rurality. Adjusted differences are for the strongly/very strongly agree comparison. Q-values represent the probability that the given difference would be a false discovery; they represent the expected proportion of “false positives” that would be seen among the collection of all differences whose q-values were at or below the given q-value.

Table S4. Association between self-reported MAGA status and expectations and perceived need for civil war in the United States

Query and Response	Republican				Non-Republican			
	MAGA Republican		Other Republican		MAGA Movement		Not MAGA Movement	
	Unweighted n	Weighted % (95% CI)	Unweighted n	Weighted % (95% CI)	Unweighted n	Weighted % (95% CI)	Unweighted n	Weighted % (95% CI)
Which of the following comes closer to your view of what a second civil war might look like?*								
Like an insurgency or guerrilla war, with small groups attacking specific targets or people.	986	85.3 (82.4, 88.2)	2364	88.1 (86.1, 90.1)	176	75.1 (66.3, 83.8)	4524	89.3 (88.0, 90.7)
Like the first Civil War in the United States, with opposing armies and large battles.	140	14.7 (11.8, 17.6)	217	11.9 (9.9, 13.9)	40	24.9 (16.2, 33.7)	342	10.7 (9.3, 12.0)
Adjusted prevalence difference (95% CI; q-value)†	7.4 (4.1, 10.6; <0.001)		3.9 (1.6, 6.3; 0.002)		11.2 (2.7, 19.8; 0.01)		Referent	
How much do you agree or disagree with each of the following statements?								
In the next few years, there will be civil war in the United States.								
Do not agree	598	51.0 (47.1, 54.8)	1852	65.5 (63.0, 68.1)	121	49.2 (39.7, 58.6)	3457	67.4 (65.6, 69.2)
Somewhat agree	450	38.0 (34.3, 41.7)	710	28.9 (26.5, 31.3)	71	33.3 (24.2, 42.4)	1308	27.8 (26.1, 29.5)
Strongly/very strongly agree	133	11.1 (8.8, 13.3)	120	5.6 (4.2, 6.9)	32	17.5 (9.9, 25.2)	192	4.8 (3.9, 5.7)
Adjusted prevalence difference (95% CI; q-value)§	7.4 (4.1, 9.8; <0.001)		2.1 (0.5, 3.7; 0.01)		12.3 (4.7, 19.9; 0.003)		Referent	
The United States needs a civil war to set things right.								
Do not agree	841	72.5 (69.2, 75.9)	2294	84.3 (82.3, 86.2)	167	73.0 (64.4, 81.6)	4626	90.2 (88.9, 91.5)
Somewhat agree	244	19.1 (16.3, 22.0)	308	11.8 (10.1, 13.4)	35	15.5 (8.4, 22.7)	250	7.0 (5.9, 8.1)
Strongly/very strongly agree	95	8.3 (6.1, 10.5)	82	4.0 (2.7, 5.2)	24	11.4 (5.3, 17.6)	93	2.8 (2.1, 3.6)
Adjusted prevalence difference (95% CI; q-value)§	6.3 (3.9, 8.6; <0.001)		1.8 (0.4, 3.2; 0.01)		7.7 (1.8, 13.5; 0.01)		Referent	

Details on assignment of MAGA status are in the Supplemental Methods Text section of this supplement (p 11).

*Q: Some people talk about a second civil war in the United States. Which of the following comes closer to your view of what a second civil war might look like? R1: A second civil war would be like the first Civil War in the United States, with opposing armies and large battles. R2: A second civil war would be like an insurgency or guerrilla war, with small groups attacking specific targets or people.

† Response options were do not agree, somewhat agree, strongly agree, very strongly agree. Findings are combined for the strongly/very strongly agree responses.

§ Adjusted models include age, race and ethnicity, gender, education, income, Census division, and rurality. Adjusted differences are for the strongly/very strongly agree comparison. Q-values represent the probability that the given difference would be a false discovery; they represent the expected proportion of “false positives” that would be seen among the collection of all differences whose q-values were at or below the given q-value.

Table S5. Association between political ideology and expectations and perceived need for civil war in the United States

Query and Response	Political Ideology							
	Extremely Liberal		Liberal		Slightly Liberal		Moderate/Middle of the Road	
	Unweighted n	Weighted % (95% CI)	Unweighted n	Weighted % (95% CI)	Unweighted n	Weighted % (95% CI)	Unweighted n	Weighted % (95% CI)
Which of the following comes closer to your view of what a second civil war might look like?*								
Like an insurgency or guerrilla war, with small groups attacking specific targets or people.	335	88.9 (83.9, 93.8)	1183	92.5 (90.0, 95.0)	779	92.8 (90.0, 95.7)	2491	86.1 (84.2, 88.0)
Like the first Civil War in the United States, with opposing armies and large battles.	26	11.1 (6.2, 16.1)	52	7.5 (5.0, 10.0)	37	7.2 (4.3, 10.0)	289	13.9 (12.0, 15.8)
Adjusted prevalence difference (95% CI; q-value)[†]	-1.0 (-6.1, 4.1; 0.79)		-4.0 (-7.0, -0.9; 0.05)		-4.3 (-7.6, -1.1; 0.05)		Referent	
How much do you agree or disagree with each of the following statements?								
In the next few years, there will be civil war in the United States.								
Do not agree	237	63.0 (56.5, 69.5)	916	71.1 (67.8, 74.4)	599	72.6 (68.5, 76.7)	1919	64.3 (61.9, 66.7)
Somewhat agree	99	30.3 (24.1, 36.5)	302	25.4 (22.2, 28.5)	198	24.1 (20.2, 28.0)	806	28.9 (26.7, 31.2)
Strongly/very strongly agree	27	6.7 (3.5, 9.9)	36	3.5 (2.1, 4.9)	26	3.3 (1.6, 4.9)	151	6.7 (5.3, 8.1)
Adjusted prevalence difference (95% CI; q-value)[§]	0.6 (-3.0, 4.2; 0.79)		-2.1 (-4.1, -0.2; 0.1)		-2.1 (-4.3, 0.1; 0.15)		Referent	
The United States needs a civil war to set things right.								
Do not agree	322	85.3 (80.4, 90.2)	1202	94.4 (92.6, 96.3)	779	91.5 (88.2, 94.7)	2568	86.9 (85.1, 88.7)
Somewhat agree	26	10.0 (5.7, 14.3)	35	3.8 (2.2, 5.4)	30	4.8 (2.6, 6.9)	234	9.3 (7.8, 10.8)
Strongly/very strongly agree	15	4.7 (1.9, 7.4)	16	1.8 (0.8, 2.7)	16	3.8 (1.3, 6.3)	85	3.8 (2.8, 4.9)
Adjusted prevalence difference (95% CI; q-value)[§]	0.9 (-2.2, 4.0; 0.75)		-1.0 (-2.4, 0.4; 0.31)		0.7 (-2.0, 3.3; 0.75)		Referent	

Table S5, continued.

Query and Response	Political Ideology					
	Slightly Conservative		Conservative		Extremely Conservative	
	Unweighted n	Weighted % (95% CI)	Unweighted n	Weighted %(95% CI)	Unweighted n	Weighted %(95% CI)
Which of the following comes closer to your view of what a second civil war might look like?*						
Like an insurgency or guerrilla war, with small groups attacking specific targets or people.	1031	90.1 (87.2, 92.9)	1816	87.1 (84.8, 89.4)	405	81.2 (75.6, 86.7)
Like the first Civil War in the United States, with opposing armies and large battles.	82	9.9 (7.1, 12.8)	192	12.9 (10.6, 15.2)	60	18.8 (13.3, 24.4)
Adjusted prevalence difference (95% CI; q-value)[†]	-1.1 (-4.3, 2.1; 0.7)		2.4 (-0.5, 5.3; 0.25)		6.5 (0.9, 12.1; 0.08)	
How much do you agree or disagree with each of the following statements?						
In the next few years, there will be civil war in the United States.						
Do not agree	810	67.8 (63.9, 71.7)	1340	60.6 (57.6, 63.6)	250	51.1 (45.0, 57.3)
Somewhat agree	300	27.3 (23.6, 31.0)	636	33.0 (30.1, 35.9)	181	37.4 (31.4, 43.3)
Strongly/very strongly agree	44	4.9 (2.9, 6.9)	124	6.4 (4.8, 8.0)	60	11.5 (7.9, 15.1)
Adjusted prevalence difference (95% CI; q-value)[§]	-0.3 (-2.7, 2.0; 0.79)		1.1 (-1.0, 3.2; 0.44)		5.5 (1.6, 9.4; 0.05)	
The United States needs a civil war to set things right.						
Do not agree	1010	85.5 (82.3, 88.8)	1739	82.0 (79.6, 84.4)	336	69.2 (63.8, 74.6)
Somewhat agree	107	10.5 (7.6, 13.3)	291	13.9 (11.8, 16.0)	113	21.9 (17.2, 26.6)
Strongly/very strongly agree	39	4.0 (2.2, 5.8)	73	4.1 (2.7, 5.5)	45	9.0 (5.6, 12.3)
Adjusted prevalence difference (95% CI; q-value)[§]	1.1 (-0.9, 3.1; 0.44)		1.2 (-0.6, 2.9; 0.35)		5.5 (2.0, 8.9; 0.03)	

Details on assignment of political ideology are in the Supplemental Methods Text section of this supplement (p 12).

*Q: Some people talk about a second civil war in the United States. Which of the following comes closer to your view of what a second civil war might look like? R1: A second civil war would be like the first Civil War in the United States, with opposing armies and large battles. R2: A second civil war would be like an insurgency or guerrilla war, with small groups attacking specific targets or people.

† Response options were do not agree, somewhat agree, strongly agree, very strongly agree. Findings are combined for the strongly/very strongly agree responses.

§ Adjusted models include age, race and ethnicity, gender, education, income, Census division, and rurality. Adjusted differences are for the strongly/very strongly agree comparison. Q-values represent the probability that the given difference would be a false discovery; they represent the expected proportion of "false positives" that would be seen among the collection of all differences whose q-values were at or below the given q-value.

Table S6. Association between racist beliefs and expectations and perceived need for civil war in the United States

Query and Response	Beliefs about Race and Ethnicity							
	Non-Agreement		Weak Agreement		Moderate Agreement		Strong Agreement	
	Unweighted n	Weighted % (95% CI)	Unweighted n	Weighted % (95% CI)	Unweighted n	Weighted % (95% CI)	Unweighted n	Weighted % (95% CI)
Which of the following comes closer to your view of what a second civil war might look like?*								
Like an insurgency or guerrilla war, with small groups attacking specific targets or people.	1593	96.5 (95.1, 97.8)	1978	90.4 (88.5, 92.4)	2611	82.5 (80.4, 84.6)	1957	86.5 (84.3, 88.8)
Like the first Civil War in the United States, with opposing armies and large battles.	40	3.5 (2.2, 4.9)	130	9.6 (7.6, 11.5)	355	17.5 (15.4, 19.6)	227	13.5 (11.2, 15.7)
Adjusted prevalence difference (95% CI; q-value)†	Referent		4.8 (2.4, 7.1; <0.001)		12.0 (9.4, 14.7; <0.001)		11.1 (8.1, 14.0; <0.001)	
How much do you agree or disagree with each of the following statements?								
In the next few years, there will be civil war in the United States.								
Do not agree	1174	70.6 (67.7, 73.5)	1631	72.8 (70.3, 75.3)	2095	62.6 (60.2, 65.0)	1234	52.9 (50.1, 55.7)
Somewhat agree	422	26.5 (23.7, 29.3)	485	24.1 (21.7, 26.5)	819	29.4 (27.1, 31.7)	843	38.0 (35.3, 40.8)
Strongly/very strongly agree	50	2.9 (1.8, 4.0)	49	3.1 (2.0, 4.2)	177	8.0 (6.5, 9.5)	201	9.1 (7.3, 10.8)
Adjusted prevalence difference (95% CI; q-value)§	Referent		-0.1 (-1.6, 1.5; 0.94)		4.7 (2.8, 6.6; <0.001)		7.1 (4.9, 9.3; <0.001)	
The United States needs a civil war to set things right.								
Do not agree	1584	94.9 (93.3, 96.4)	2094	95.8 (94.6, 97.0)	2683	81.9 (79.8, 84.0)	1689	73.4 (70.8, 76.0)
Somewhat agree	52	4.0 (2.7, 5.4)	65	3.3 (2.2, 4.4)	287	12.2 (10.4, 13.9)	443	19.6 (17.3, 21.8)
Strongly/very strongly agree	13	1.1 (0.4, 1.8)	12	0.9 (0.3, 1.5)	123	5.9 (4.6, 7.3)	148	7.1 (5.4, 8.7)
Adjusted prevalence difference (95% CI; q-value)§	Referent		-0.4 (-1.3, 0.5; 0.48)		4.4 (2.8, 5.9; <0.001)		6.5 (4.6, 8.4; <0.001)	

Details on assignment of agreement with beliefs about race and ethnicity are in the Supplemental Methods Text section of this supplement (p 12).

*Q: Some people talk about a second civil war in the United States. Which of the following comes closer to your view of what a second civil war might look like? R1: A second civil war would be like the first Civil War in the United States, with opposing armies and large battles. R2: A second civil war would be like an insurgency or guerrilla war, with small groups attacking specific targets or people.

† Response options were do not agree, somewhat agree, strongly agree, very strongly agree. Findings are combined for the strongly/very strongly agree responses.

§ Adjusted models include age, race and ethnicity, gender, education, income, Census division, and rurality. Adjusted differences are for the strongly/very strongly agree comparison. Q-values represent the probability that the given difference would be a false discovery; they represent the expected proportion of “false positives” that would be seen among the collection of all differences whose q-values were at or below the given q-value.

Table S7. Association between pro-violence beliefs and expectations and perceived need for civil war in the United States

Query and Response	Beliefs about Violence to Effect Social Change							
	Non-Agreement		Weak Agreement		Moderate Agreement		Strong Agreement	
	Unweighted n	Weighted % (95% CI)	Unweighted n	Weighted % (95% CI)	Unweighted n	Weighted % (95% CI)	Unweighted n	Weighted % (95% CI)
Which of the following comes closer to your view of what a second civil war might look like?*								
Like an insurgency or guerrilla war, with small groups attacking specific targets or people.	4332	93.3 (92.2, 94.4)	2107	89.7 (87.8, 91.6)	1188	76.4 (73, 79.8)	524	75.4 (70.3, 80.5)
Like the first Civil War in the United States, with opposing armies and large battles.	206	6.7 (5.6, 7.8)	190	10.3 (8.4, 12.2)	237	23.6 (20.2, 27)	119	24.6 (19.5, 29.7)
Adjusted prevalence difference (95% CI; q-value)[†]	Referent		2.2 (0.1, 4.4; 0.04)		13.8 (10.3, 17.3; <0.001)		14.3 (9.3, 19.3; <0.001)	
How much do you agree or disagree with each of the following statements?								
In the next few years, there will be civil war in the United States.								
Do not agree	3761	79.4 (77.8, 80.9)	1593	65.0 (62.4, 67.7)	644	40.0 (36.5, 43.5)	156	25.3 (20.6, 30.1)
Somewhat agree	864	18.8 (17.3, 20.3)	701	30.4 (27.9, 32.9)	702	51.1 (47.4, 54.8)	299	43.2 (37.9, 48.4)
Strongly/very strongly agree	80	1.8 (1.3, 2.4)	77	4.6 (3.2, 5.9)	118	8.9 (6.8, 11.0)	202	31.5 (26.5, 36.6)
Adjusted prevalence difference (95% CI; q-value)[§]	Referent		2.3 (0.9, 3.8; 0.002)		6.1 (4.0, 8.2; <0.001)		28.3 (23.3, 33.2; <0.001)	
The United States needs a civil war to set things right.								
Do not agree	4640	98.2 (97.7, 98.7)	2191	91.9 (90.4, 93.4)	981	62.0 (58.3, 65.7)	260	42.8 (37.5, 48.2)
Somewhat agree	66	1.5 (1.0, 1.9)	161	6.6 (5.3, 7.9)	400	31.2 (27.6, 34.7)	222	27.2 (22.9, 31.6)
Strongly/very strongly agree	14	0.3 (0.1, 0.6)	23	1.5 (0.7, 2.3)	82	6.8 (4.8, 8.8)	177	29.9 (24.9, 35.0)
Adjusted prevalence difference (95% CI; q-value)[§]	Referent		0.8 (0.0, 1.6; 0.04)		5.7 (3.8, 7.7; <0.001)		28.3 (23.4, 33.1; <0.001)	

Details on assignment of agreement with beliefs about violence to effect social change are in the Supplemental Methods Text section of this supplement (p 12).

*Q: Some people talk about a second civil war in the United States. Which of the following comes closer to your view of what a second civil war might look like? R1: A second civil war would be like the first Civil War in the United States, with opposing armies and large battles. R2: A second civil war would be like an insurgency or guerrilla war, with small groups attacking specific targets or people.

† Response options were do not agree, somewhat agree, strongly agree, very strongly agree. Findings are combined for the strongly/very strongly agree responses.

§ Adjusted models include age, race and ethnicity, gender, education, income, Census division, and rurality. Adjusted differences are for the strongly/very strongly agree comparison. Q-values represent the probability that the given difference would be a false discovery; they represent the expected proportion of “false positives” that would be seen among the collection of all differences whose q-values were at or below the given q-value.

Table S8. Association between approval of extreme right-wing organizations and movements and expectations and perceived need for civil war in the United States

Query and Response	Approval of Organizations and Movements							
	Non-Approval		Weak Approval		Moderate Approval		Strong Approval	
	Unweighted n	Weighted % (95% CI)	Unweighted n	Weighted % (95% CI)	Unweighted n	Weighted % (95% CI)	Unweighted n	Weighted % (95% CI)
Which of the following comes closer to your view of what a second civil war might look like?*								
Like an insurgency or guerrilla war, with small groups attacking specific targets or people.	2563	94.2 (93.0, 95.4)	437	82.0 (77.2, 86.8)	123	76.3 (66.5, 86.0)	29	51.9 (33.7, 70.2)
Like the first Civil War in the United States, with opposing armies and large battles.	128	5.8 (4.6, 7.0)	72	18.0 (13.2, 22.8)	26	23.7 (14.0, 33.5)	19	48.1 (29.8, 66.3)
Adjusted prevalence difference (95% CI; q-value)[†]	Referent		10.3 (5.3, 15.2; <0.001)		11.8 (2.9, 20.8; 0.01)		34.5 (17.5, 51.6; <0.001)	
How much do you agree or disagree with each of the following statements?								
In the next few years, there will be civil war in the United States.								
Do not agree	2030	72.2 (70.1, 74.3)	291	55.0 (49.3, 60.7)	56	29.9 (20.7, 39.0)	10	24.7 (8.3, 41.2)
Somewhat agree	632	24.7 (22.6, 26.7)	199	37.0 (31.5, 42.5)	71	49.7 (39.1, 60.4)	14	35.0 (17.4, 52.7)
Strongly/very strongly agree	79	3.1 (2.2, 4.0)	41	8.0 (4.7, 11.3)	29	20.4 (11.6, 29.3)	25	40.2 (23.2, 57.2)
Adjusted prevalence difference (95% CI; q-value)[§]	Referent		3.7 (0.5, 6.9; 0.02)		14.9 (6.4, 23.5; 0.001)		33.5 (16.2, 50.8; <0.001)	
The United States needs a civil war to set things right.								
Do not agree	2616	93.8 (92.5, 95.1)	421	79.0 (74.2, 83.8)	83	43.3 (33.1, 53.6)	14	32.2 (15.5, 48.9)
Somewhat agree	103	4.8 (3.7, 6.0)	74	14.0 (9.7, 18.2)	48	37.1 (26.3, 47.9)	11	27.6 (11.5, 43.7)
Strongly/very strongly agree	27	1.3 (0.8, 1.9)	35	7.0 (4.2, 9.9)	25	19.6 (10.8, 28.4)	24	40.2 (22.5, 57.9)
Adjusted prevalence difference (95% CI; q-value)[§]	Referent		4.6 (1.8, 7.4; 0.002)		16.4 (8.0, 24.8; <0.001)		36.7 (19.7, 53.6; <0.001)	

Details on assignment of approval of organizations and movements are in the Supplemental Methods Text section of this supplement (p 12).

*Q: Some people talk about a second civil war in the United States. Which of the following comes closer to your view of what a second civil war might look like? R1: A second civil war would be like the first Civil War in the United States, with opposing armies and large battles. R2: A second civil war would be like an insurgency or guerrilla war, with small groups attacking specific targets or people.

† Response options were do not agree, somewhat agree, strongly agree, very strongly agree. Findings are combined for the strongly/very strongly agree responses.

§ Adjusted models include age, race and ethnicity, gender, education, income, Census division, and rurality. Adjusted differences are for the strongly/very strongly agree comparison. Q-values represent the probability that the given difference would be a false discovery; they represent the expected proportion of “false positives” that would be seen among the collection of all differences whose q-values were at or below the given q-value.

Table S9. Association between approval of specific extreme right-wing organizations and movements and expectations and perceived need for civil war in the United States

Query and Response	Proud Boys					
	Do Not Approve		Somewhat Approve		Strongly/Very Strongly Approve	
	Unweighted n	Weighted % (95% CI)	Unweighted n	Weighted % (95% CI)	Unweighted n	Weighted % (95% CI)
Which of the following comes closer to your view of what a second civil war might look like?*						
Like an insurgency or guerrilla war, with small groups attacking specific targets or people.	5104	93.7 (92.8, 94.6)	327	82.4 (75.7, 89.2)	114	66.2 (55.4, 77.0)
Like the first Civil War in the United States, with opposing armies and large battles.	277	6.3 (5.4, 7.2)	39	17.6 (10.8, 24.3)	40	33.8 (23.0, 44.6)
Adjusted prevalence difference (95% CI; q-value)†	Referent		9.0 (2.7, 15.3; 0.006)		23.7 (13.4, 34.0; <0.001)	
How much do you agree or disagree with each of the following statements?						
In the next few years, there will be civil war in the United States.						
Do not agree	3904	70.0 (68.3, 71.6)	178	42.3 (35.1, 49.6)	58	38.3 (27.7, 49.0)
Somewhat agree	1400	26.3 (24.7, 27.9)	158	46.3 (38.9, 53.8)	45	30.7 (20.2, 41.1)
Strongly/very strongly agree	185	3.7 (3.0, 4.5)	40	11.3 (6.7, 15.9)	52	31.0 (21.1, 40.9)
Adjusted prevalence difference (95% CI; q-value)§	Referent		6.6 (1.8, 11.3; 0.007)		25.1 (15.2, 35.1; <0.001)	
The United States needs a civil war to set things right.						
Do not agree	5127	92.4 (91.4, 93.5)	255	61.1 (53.6, 68.6)	71	45.6 (34.7, 56.5)
Somewhat agree	289	5.8 (4.9, 6.7)	87	29.9 (22.5, 37.2)	38	23.2 (14.6, 31.8)
Strongly/very strongly agree	80	1.8 (1.3, 2.3)	34	9.1 (5.0, 13.1)	47	31.2 (20.7, 41.8)
Adjusted prevalence difference (95% CI; q-value)§	Referent		6.2 (1.9, 10.4; 0.006)		27.4 (17.3, 37.4; <0.001)	

Table S9, continued.

Query and Response	Oath Keepers					
	Do Not Approve		Somewhat Approve		Strongly/Very Strongly Approve	
	Unweighted n	Weighted % (95% CI)	Unweighted n	Weighted % (95% CI)	Unweighted n	Weighted % (95% CI)
Which of the following comes closer to your view of what a second civil war might look like?*						
Like an insurgency or guerrilla war, with small groups attacking specific targets or people.	3788	93.7 (92.5, 94.9)	317	81.9 (74.8, 88.9)	161	68.8 (59, 78.5)
Like the first Civil War in the United States, with opposing armies and large battles.	185	6.3 (5.1, 7.5)	36	18.1 (11.1, 25.2)	51	31.2 (21.5, 41.0)
Adjusted prevalence difference (95% CI; q-value)[†]	Referent		8.9 (2.0, 15.8; 0.02)		20.3 (11.2, 29.4; <0.001)	
How much do you agree or disagree with each of the following statements?						
In the next few years, there will be civil war in the United States.						
Do not agree	2911	70.6 (68.7, 72.5)	207	53.4 (45.9, 60.9)	83	32.7 (24.1, 41.3)
Somewhat agree	1006	25.9 (24.1, 27.8)	131	38.3 (30.9, 45.6)	69	31.5 (22.0, 41.0)
Strongly/very strongly agree	133	3.5 (2.6, 4.3)	30	8.3 (3.7, 13.0)	68	35.8 (26.4, 45.2)
Adjusted prevalence difference (95% CI; q-value)[§]	Referent		3.3 (-1.0, 7.6; 0.13)		28.8 (19.5, 38.1; <0.001)	
The United States needs a civil war to set things right.						
Do not agree	3805	92.6 (91.3, 93.8)	272	67.1 (59.3, 74.9)	115	47.5 (37.9, 57.1)
Somewhat agree	196	5.7 (4.5, 6.8)	75	26.0 (18.6, 33.4)	44	16.9 (9.9, 24.0)
Strongly/very strongly agree	54	1.7 (1.2, 2.3)	21	6.9 (2.4, 11.4)	62	35.6 (25.8, 45.4)
Adjusted prevalence difference (95% CI; q-value)[§]	Referent		3.5 (-0.6, 7.5; 0.11)		30.7 (21.7, 39.8; <0.001)	

Table S9, continued.

Query and Response	Three Percenters					
	Do Not Approve		Somewhat Approve		Strongly/Very Strongly Approve	
	Unweighted n	Weighted % (95% CI)	Unweighted n	Weighted % (95% CI)	Unweighted n	Weighted % (95% CI)
Which of the following comes closer to your view of what a second civil war might look like?*						
Like an insurgency or guerrilla war, with small groups attacking specific targets or people.	2635	92 (90.3, 93.6)	171	81.8 (73.4, 90.2)	67	60.5 (46.9, 74.2)
Like the first Civil War in the United States, with opposing armies and large battles.	154	8.0 (6.4, 9.7)	24	18.2 (9.8, 26.6)	32	39.5 (25.8, 53.1)
Adjusted prevalence difference (95% CI; q-value)[†]	Referent		5.9 (-2.7, 14.5; 0.18)		26.1 (12.9, 39.2; <0.001)	
How much do you agree or disagree with each of the following statements?						
In the next few years, there will be civil war in the United States.						
Do not agree	1988	67.3 (64.9, 69.7)	86	45.7 (36.0, 55.5)	39	34.7 (22.0, 47.4)
Somewhat agree	757	28.7 (26.3, 31.0)	84	42.8 (33.2, 52.3)	25	25.5 (13.1, 37.9)
Strongly/very strongly agree	110	4.1 (3.0, 5.1)	27	11.5 (5.2, 17.8)	39	39.8 (26.5, 53.0)
Adjusted prevalence difference (95% CI; q-value)[§]	Referent		5.3 (-0.7, 11.3; 0.1)		33.3 (20.1, 46.5; <0.001)	
The United States needs a civil war to set things right.						
Do not agree	2630	90.0 (88.3, 91.8)	125	58.4 (48.4, 68.3)	60	49.7 (36.2, 63.1)
Somewhat agree	175	7.4 (5.8, 8.9)	49	28.4 (18.9, 37.8)	13	14.2 (5.2, 23.2)
Strongly/very strongly agree	53	2.6 (1.8, 3.4)	23	13.3 (6.0, 20.5)	31	36.1 (22.6, 49.6)
Adjusted prevalence difference (95% CI; q-value)[§]	Referent		7.4 (1.0, 13.8; 0.04)		30.7 (18.1, 43.3; <0.001)	

Table S9, continued.

Query and Response	QAnon					
	Do Not Approve		Somewhat Approve		Strongly/Very Strongly Approve	
	Unweighted n	Weighted % (95% CI)	Unweighted n	Weighted % (95% CI)	Unweighted n	Weighted % (95% CI)
Which of the following comes closer to your view of what a second civil war might look like?*						
Like an insurgency or guerrilla war, with small groups attacking specific targets or people.	4982	93.6 (92.6, 94.6)	173	77.0 (68.0, 86.0)	72	65.4 (51.7, 79.2)
Like the first Civil War in the United States, with opposing armies and large battles.	256	6.4 (5.4, 7.4)	31	23.0 (14.0, 32.0)	26	34.6 (20.8, 48.3)
Adjusted prevalence difference (95% CI; q-value)[†]	Referent		13.1 (4.8, 21.5; 0.003)		21.9 (8.7, 35.1; 0.002)	
How much do you agree or disagree with each of the following statements?						
In the next few years, there will be civil war in the United States.						
Do not agree	3793	70.2 (68.5, 71.8)	97	42.4 (33.3, 51.5)	39	33.7 (20.8, 46.5)
Somewhat agree	1359	26.2 (24.6, 27.8)	83	43.1 (33.8, 52.5)	27	31.8 (18.5, 45.0)
Strongly/very strongly agree	192	3.6 (2.9, 4.3)	30	14.5 (7.4, 21.5)	33	34.6 (21.4, 47.8)
Adjusted prevalence difference (95% CI; q-value)[§]	Referent		7.7 (1.5, 13.8; 0.02)		28.5 (15.2, 41.8; <0.001)	
The United States needs a civil war to set things right.						
Do not agree	4936	91.5 (90.5, 92.6)	142	60.3 (50.8, 69.8)	49	40.0 (27.1, 53.0)
Somewhat agree	320	6.4 (5.5, 7.4)	48	28.4 (19.5, 37.4)	22	27.0 (14.2, 39.7)
Strongly/very strongly agree	96	2.0 (1.5, 2.6)	21	11.3 (4.6, 17.9)	29	33.0 (19.5, 46.4)
Adjusted prevalence difference (95% CI; q-value)[§]	Referent		5.8 (0.3, 11.4; 0.04)		29.2 (16.5, 41.9; <0.001)	

Table S9, continued.

Query and Response	Christian Nationalist Movement					
	Do Not Approve		Somewhat Approve		Strongly/Very Strongly Approve	
	Unweighted n	Weighted % (95% CI)	Unweighted n	Weighted % (95% CI)	Unweighted n	Weighted % (95% CI)
Which of the following comes closer to your view of what a second civil war might look like?*						
Like an insurgency or guerrilla war, with small groups attacking specific targets or people.	5104	93.7 (92.8, 94.6)	327	82.4 (75.7, 89.2)	114	66.2 (55.4, 77.0)
Like the first Civil War in the United States, with opposing armies and large battles.	277	6.3 (5.4, 7.2)	39	17.6 (10.8, 24.3)	40	33.8 (23.0, 44.6)
Adjusted prevalence difference (95% CI; q-value)[†]	Referent		5.7 (1.2, 10.3; 0.02)		21.4 (14.4, 28.3; <0.001)	
How much do you agree or disagree with each of the following statements?						
In the next few years, there will be civil war in the United States.						
Do not agree	3904	70.0 (68.3, 71.6)	178	42.3 (35.1, 49.6)	58	38.3 (27.7, 49.0)
Somewhat agree	1400	26.3 (24.7, 27.9)	158	46.3 (38.9, 53.8)	45	30.7 (20.2, 41.1)
Strongly/very strongly agree	185	3.7 (3.0, 4.5)	40	11.3 (6.7, 15.9)	52	31.0 (21.1, 40.9)
Adjusted prevalence difference (95% CI; q-value)[§]	Referent		2.4 (-0.9, 5.6; 0.16)		18.1 (12.0, 24.3; <0.001)	
The United States needs a civil war to set things right.						
Do not agree	5127	92.4 (91.4, 93.5)	255	61.1 (53.6, 68.6)	71	45.6 (34.7, 56.5)
Somewhat agree	289	5.8 (4.9, 6.7)	87	29.9 (22.5, 37.2)	38	23.2 (14.6, 31.8)
Strongly/very strongly agree	80	1.8 (1.3, 2.3)	34	9.1 (5.0, 13.1)	47	31.2 (20.7, 41.8)
Adjusted prevalence difference (95% CI; q-value)[§]	Referent		2.9 (0.3, 5.6; 0.03)		18.1 (12.2, 24.0; <0.001)	

Table S9, continued.

Query and Response	White Supremacy Movement					
	Do Not Approve		Somewhat Approve		Strongly/Very Strongly Approve	
	Unweighted n	Weighted % (95% CI)	Unweighted n	Weighted % (95% CI)	Unweighted n	Weighted % (95% CI)
Which of the following comes closer to your view of what a second civil war might look like?*						
Like an insurgency or guerrilla war, with small groups attacking specific targets or people.	3788	93.7 (92.5, 94.9)	317	81.9 (74.8, 88.9)	161	68.8 (59.0, 78.5)
Like the first Civil War in the United States, with opposing armies and large battles.	185	6.3 (5.1, 7.5)	36	18.1 (11.1, 25.2)	51	31.2 (21.5, 41.0)
Adjusted prevalence difference (95% CI; q-value)[†]	Referent		10.9 (-0.6, 22.3; 0.06)		21.9 (5.3, 38.5; 0.01)	
How much do you agree or disagree with each of the following statements?						
In the next few years, there will be civil war in the United States.						
Do not agree	2911	70.6 (68.7, 72.5)	207	53.4 (45.9, 60.9)	83	32.7 (24.1, 41.3)
Somewhat agree	1006	25.9 (24.1, 27.8)	131	38.3 (30.9, 45.6)	69	31.5 (22.0, 41.0)
Strongly/very strongly agree	133	3.5 (2.6, 4.3)	30	8.3 (3.7, 13.0)	68	35.8 (26.4, 45.2)
Adjusted prevalence difference (95% CI; q-value)[§]	Referent		11.0 (1.7, 20.3; 0.02)		32.1 (16.1, 48.1; <0.001)	
The United States needs a civil war to set things right.						
Do not agree	3805	92.6 (91.3, 93.8)	272	67.1 (59.3, 74.9)	115	47.5 (37.9, 57.1)
Somewhat agree	196	5.7 (4.5, 6.8)	75	26.0 (18.6, 33.4)	44	16.9 (9.9, 24.0)
Strongly/very strongly agree	54	1.7 (1.2, 2.3)	21	6.9 (2.4, 11.4)	62	35.6 (25.8, 45.4)
Adjusted prevalence difference (95% CI; q-value)[§]	Referent		15.1 (4.6, 25.7; 0.009)		37.2 (21.2, 53.2; <0.001)	

Table S9, continued.

Query and Response	Militia Movement					
	Do Not Approve		Somewhat Approve		Strongly/Very Strongly Approve	
	Unweighted n	Weighted % (95% CI)	Unweighted n	Weighted % (95% CI)	Unweighted n	Weighted % (95% CI)
Which of the following comes closer to your view of what a second civil war might look like?*						
Like an insurgency or guerrilla war, with small groups attacking specific targets or people.	5104	93.7 (92.8, 94.6)	327	82.4 (75.7, 89.2)	114	66.2 (55.4, 77.0)
Like the first Civil War in the United States, with opposing armies and large battles.	277	6.3 (5.4, 7.2)	39	17.6 (10.8, 24.3)	40	33.8 (23.0, 44.6)
Adjusted prevalence difference (95% CI; q-value)[†]	Referent		5.2 (-0.5, 10.9; 0.07)		25.3 (13.9, 36.7; <0.001)	
How much do you agree or disagree with each of the following statements?						
In the next few years, there will be civil war in the United States.						
Do not agree	3904	70.0 (68.3, 71.6)	178	42.3 (35.1, 49.6)	58	38.3 (27.7, 49.0)
Somewhat agree	1400	26.3 (24.7, 27.9)	158	46.3 (38.9, 53.8)	45	30.7 (20.2, 41.1)
Strongly/very strongly agree	185	3.7 (3.0, 4.5)	40	11.3 (6.7, 15.9)	52	31.0 (21.1, 40.9)
Adjusted prevalence difference (95% CI; q-value)[§]	Referent		4.1 (0.0, 8.2; 0.07)		21.2 (10.7, 31.7; <0.001)	
The United States needs a civil war to set things right.						
Do not agree	5127	92.4 (91.4, 93.5)	255	61.1 (53.6, 68.6)	71	45.6 (34.7, 56.5)
Somewhat agree	289	5.8 (4.9, 6.7)	87	29.9 (22.5, 37.2)	38	23.2 (14.6, 31.8)
Strongly/very strongly agree	80	1.8 (1.3, 2.3)	34	9.1 (5.0, 13.1)	47	31.2 (20.7, 41.8)
Adjusted prevalence difference (95% CI; q-value)[§]	Referent		3.6 (-0.2, 7.4; 0.07)		25.2 (14.8, 35.6; <0.001)	

Table S9, continued.

Query and Response	Boogaloo Movement					
	Do Not Approve		Somewhat Approve		Strongly/Very Strongly Approve	
	Unweighted n	Weighted % (95% CI)	Unweighted n	Weighted % (95% CI)	Unweighted n	Weighted % (95% CI)
Which of the following comes closer to your view of what a second civil war might look like?*						
Like an insurgency or guerrilla war, with small groups attacking specific targets or people.	3788	93.7 (92.5, 94.9)	317	81.9 (74.8, 88.9)	161	68.8 (59.0, 78.5)
Like the first Civil War in the United States, with opposing armies and large battles.	185	6.3 (5.1, 7.5)	36	18.1 (11.1, 25.2)	51	31.2 (21.5, 41.0)
Adjusted prevalence difference (95% CI; q-value)†	Referent		5.5 (-5.2, 16.2; 0.31)		25.9 (7.8, 44.0; 0.01)	
How much do you agree or disagree with each of the following statements?						
In the next few years, there will be civil war in the United States.						
Do not agree	2911	70.6 (68.7, 72.5)	207	53.4 (45.9, 60.9)	83	32.7 (24.1, 41.3)
Somewhat agree	1006	25.9 (24.1, 27.8)	131	38.3 (30.9, 45.6)	69	31.5 (22.0, 41.0)
Strongly/very strongly agree	133	3.5 (2.6, 4.3)	30	8.3 (3.7, 13.0)	68	35.8 (26.4, 45.2)
Adjusted prevalence difference (95% CI; q-value)§	Referent		16.3 (5.2, 27.4; 0.01)		22.0 (6.2, 37.8; 0.01)	
The United States needs a civil war to set things right.						
Do not agree	3805	92.6 (91.3, 93.8)	272	67.1 (59.3, 74.9)	115	47.5 (37.9, 57.1)
Somewhat agree	196	5.7 (4.5, 6.8)	75	26.0 (18.6, 33.4)	44	16.9 (9.9, 24.0)
Strongly/very strongly agree	54	1.7 (1.2, 2.3)	21	6.9 (2.4, 11.4)	62	35.6 (25.8, 45.4)
Adjusted prevalence difference (95% CI; q-value)§	Referent		13.8 (3.5, 24.1; 0.01)		35.1 (18.7, 51.5; <0.001)	

*Q: Some people talk about a second civil war in the United States. Which of the following comes closer to your view of what a second civil war might look like? R1: A second civil war would be like the first Civil War in the United States, with opposing armies and large battles. R2: A second civil war would be like an insurgency or guerrilla war, with small groups attacking specific targets or people.

† Response options were do not agree, somewhat agree, strongly agree, very strongly agree. Findings are combined for the strongly/very strongly agree responses.

§ Adjusted models include age, race and ethnicity, gender, education, income, Census division, and rurality. Adjusted differences are for the strongly/very strongly agree comparison. Q-values represent the probability that the given difference would be a false discovery; they represent the expected proportion of "false positives" that would be seen among the collection of all differences whose q-values were at or below the given q-value.

Table S10. Association between firearm ownership status and expectations and perceived need for civil war in the United States

Query and Response	Firearm Ownership Status					
	Nonowner without Firearms at Home		Nonowner with Firearms at Home		Owner	
	Unweighted n	Weighted % (95% CI)	Unweighted n	Weighted % (95% CI)	Unweighted n	Weighted % (95% CI)
Which of the following comes closer to your view of what a second civil war might look like?*						
Like an insurgency or guerrilla war, with small groups attacking specific targets or people.	3683	87.7 (86.3, 89.1)	543	87.3 (83.5, 91.1)	3890	89.7 (88.2, 91.2)
Like the first Civil War in the United States, with opposing armies and large battles.	376	12.3 (10.9, 13.7)	56	12.7 (8.9, 16.5)	318	10.3 (8.8, 11.8)
Adjusted prevalence difference (95% CI; q-value)†	Referent		1.1 (-2.9, 5.0; 0.71)		1.8 (-0.3, 3.9; 0.19)	
How much do you agree or disagree with each of the following statements?						
In the next few years, there will be civil war in the United States.						
Do not agree	2884	65.8 (64.0, 67.6)	400	65.0 (60.4, 69.6)	2841	62.8 (60.9, 64.7)
Somewhat agree	1125	28.3 (26.6, 30.1)	187	30.6 (26.2, 35.0)	1244	30.4 (28.6, 32.2)
Strongly/very strongly agree	198	5.9 (4.9, 6.9)	26	4.3 (2.3, 6.4)	255	6.8 (5.7, 7.8)
Adjusted prevalence difference (95% CI; q-value)§	Referent		-0.9 (-3.2, 1.3; 0.63)		2.5 (1.0, 4.0; 0.005)	
The United States needs a civil war to set things right.						
Do not agree	3792	87.5 (86.1, 88.9)	542	88.1 (84.7, 91.4)	3705	83.2 (81.7, 84.7)
Somewhat agree	309	8.7 (7.5, 9.9)	59	9.0 (6.2, 11.8)	478	12.3 (11.0, 13.7)
Strongly/very strongly agree	121	3.8 (3.0, 4.7)	13	2.9 (1.0, 4.9)	163	4.4 (3.6, 5.3)
Adjusted prevalence difference (95% CI; q-value)§	Referent		0.3 (-1.8, 2.4; 0.78)		1.5 (0.3, 2.6; 0.04)	

Details on assignment of firearm ownership status are in the Supplemental Methods Text section of this supplement (p 13).

*Q: Some people talk about a second civil war in the United States. Which of the following comes closer to your view of what a second civil war might look like? R1: A second civil war would be like the first Civil War in the United States, with opposing armies and large battles. R2: A second civil war would be like an insurgency or guerrilla war, with small groups attacking specific targets or people.

† Response options were do not agree, somewhat agree, strongly agree, very strongly agree. Findings are combined for the strongly/very strongly agree responses.

§ Adjusted models include age, race and ethnicity, gender, education, income, Census division, and rurality. Adjusted differences are for the strongly/very strongly agree comparison. Q-values represent the probability that the given difference would be a false discovery; they represent the expected proportion of "false positives" that would be seen among the collection of all differences whose q-values were at or below the given q-value.

Table S11. Association between types(s) of firearm owned and expectations and perceived need for civil war in the United States

Query and Response	Type(s) of Firearm Owned							
	Handgun Only		Other		Other Rifle		Assault-Type Rifle	
	Unweighted n	Weighted % (95% CI)	Unweighted n	Weighted % (95% CI)	Unweighted n	Weighted % (95% CI)	Unweighted n	Weighted % (95% CI)
Which of the following comes closer to your view of what a second civil war might look like?*								
Like an insurgency or guerrilla war, with small groups attacking specific targets or people.	916	89.1 (86.4, 91.8)	499	93.6 (91.2, 95.9)	1706	90.4 (87.8, 93.0)	694	86.8 (83.0, 90.6)
Like the first Civil War in the United States, with opposing armies and large battles.	81	10.9 (8.2, 13.6)	34	6.4 (4.1, 8.8)	123	9.6 (7.0, 12.2)	69	13.2 (9.4, 17.0)
Adjusted prevalence difference (95% CI; q-value)†	Referent		-0.8 (-4.4, 2.8; 0.66)		1.8 (-1.9, 5.5; 0.42)		5.5 (1.1, 9.8; 0.04)	
How much do you agree or disagree with each of the following statements?								
In the next few years, there will be civil war in the United States.								
Do not agree	674	64.3 (60.6, 67.9)	377	65.4 (60.4, 70.4)	1263	64.5 (61.5, 67.5)	463	56.8 (52.4, 61.2)
Somewhat agree	305	30.5 (27.0, 34.1)	143	26.6 (22.2, 31.1)	538	30.1 (27.2, 33.0)	239	33.2 (28.9, 37.6)
Strongly/very strongly agree	49	5.2 (3.5, 6.9)	28	8.0 (4.6, 11.4)	87	5.4 (3.8, 7.0)	82	10.0 (7.5, 12.5)
Adjusted prevalence difference (95% CI; q-value)§	Referent		3.7 (-0.2, 7.6; 0.11)		1.2 (-1.4, 3.7; 0.42)		5.2 (2.0, 8.3; 0.01)	
The United States needs a civil war to set things right.								
Do not agree	912	86.8 (84.2, 89.5)	481	84.3 (80.2, 88.4)	1641	85.5 (83.3, 87.7)	597	75.3 (71.5, 79.1)
Somewhat agree	96	10.5 (8.1, 12.9)	45	9.7 (6.6, 12.8)	184	10.5 (8.6, 12.4)	139	18.2 (14.8, 21.6)
Strongly/very strongly agree	24	2.7 (1.5, 3.8)	22	6.1 (3.0, 9.1)	64	4.0 (2.7, 5.3)	48	6.4 (4.3, 8.6)
Adjusted prevalence difference (95% CI; q-value)§	Referent		3.6 (0.2, 7.0; 0.09)		1.7 (-0.3, 3.7; 0.16)		3.7 (1.0, 6.3; 0.03)	

Details on classification of firearm owners by type(s) of firearms owned are in the Supplemental Methods Text section of this supplement (p 13).

*Q: Some people talk about a second civil war in the United States. Which of the following comes closer to your view of what a second civil war might look like? R1: A second civil war would be like the first Civil War in the United States, with opposing armies and large battles. R2: A second civil war would be like an insurgency or guerrilla war, with small groups attacking specific targets or people.

† Response options were do not agree, somewhat agree, strongly agree, very strongly agree. Findings are combined for the strongly/very strongly agree responses.

§ Adjusted models include age, race and ethnicity, gender, education, income, Census division, and rurality. Adjusted differences are for the strongly/very strongly agree comparison. Q-values represent the probability that the given difference would be a false discovery; they represent the expected proportion of “false positives” that would be seen among the collection of all differences whose q-values were at or below the given q-value.

Table S12. Association between recency of firearm purchase and expectations and perceived need for civil war in the United States

Query and Response	Recency of Firearm Purchase			
	Purchases Only 2019 or Earlier		Purchases 2020 or Later	
	Unweighted n	Weighted % (95% CI)	Unweighted n	Weighted % (95% CI)
Which of the following comes closer to your view of what a second civil war might look like?*				
Like an insurgency or guerrilla war, with small groups attacking specific targets or people.	2771	91.1 (89.4, 92.8)	1072	87.1 (84.2, 90.0)
Like the first Civil War in the United States, with opposing armies and large battles.	201	8.9 (7.2, 10.6)	111	12.9 (10.0, 15.8)
Adjusted prevalence difference (95% CI; q-value)†	Referent		3.7 (0.7, 6.7; 0.02)	
How much do you agree or disagree with each of the following statements?				
In the next few years, there will be civil war in the United States.				
Do not agree	2080	65.5 (63.2, 67.8)	725	57.1 (53.6, 60.6)
Somewhat agree	841	28.7 (26.5, 30.9)	388	34.2 (30.8, 37.6)
Strongly/very strongly agree	146	5.8 (4.6, 7.0)	105	8.7 (6.7, 10.6)
Adjusted prevalence difference (95% CI; q-value)§	Referent		2.5 (0.0, 4.9; 0.05)	
The United States needs a civil war to set things right.				
Do not agree	2706	86.2 (84.4, 87.9)	952	76.8 (73.8, 79.8)
Somewhat agree	296	10.9 (9.3, 12.5)	180	15.9 (13.3, 18.5)
Strongly/very strongly agree	74	3.0 (2.1, 3.8)	82	7.3 (5.5, 9.1)
Adjusted prevalence difference (95% CI; q-value)§	Referent		3.9 (1.8, 5.9; 0.001)	

Details on classification of firearm owners by recency of purchase are in the Supplemental Methods Text section of this supplement (p 13).

*Q: Some people talk about a second civil war in the United States. Which of the following comes closer to your view of what a second civil war might look like? R1: A second civil war would be like the first Civil War in the United States, with opposing armies and large battles. R2: A second civil war would be like an insurgency or guerrilla war, with small groups attacking specific targets or people.

† Response options were do not agree, somewhat agree, strongly agree, very strongly agree. Findings are combined for the strongly/very strongly agree responses.

§ Adjusted models include age, race and ethnicity, gender, education, income, Census division, and rurality. Adjusted differences are for the strongly/very strongly agree comparison. Q-values represent the probability that the given difference would be a false discovery; they represent the expected proportion of “false positives” that would be seen among the collection of all differences whose q-values were at or below the given q-value.

Table S13. Association between frequency of firearm carrying and expectations and perceived need for civil war in the United States

**Query and Response**	**Carrying Loaded Firearm When Out in Public in the Past Year**					
	**Never or Not Often at All**		**Less Than Half, About Half, or More Than Half the Time**		**All or Nearly All the Time**	
	**Unweighted n**	**Weighted % (95% CI)**	**Unweighted n**	**Weighted % (95% CI)**	**Unweighted n**	**Weighted % (95% CI)**
Which of the following comes closer to your view of what a second civil war might look like?*						
Like an insurgency or guerrilla war, with small groups attacking specific targets or people.	3071	90.7 (89.1, 92.3)	512	88.7 (85.3, 92.1)	295	82.8 (75.2, 90.4)
Like the first Civil War in the United States, with opposing armies and large battles.	229	9.3 (7.7, 10.9)	54	11.3 (7.9, 14.7)	34	17.2 (9.6, 24.8)
Adjusted prevalence difference (95% CI; q-value)†	Referent		1.5 (-2.3, 5.3; 0.44)		5.7 (0.0, 11.4; 0.06)	
How much do you agree or disagree with each of the following statements?						
In the next few years, there will be civil war in the United States.						
Do not agree	2335	65.9 (63.8, 68.1)	329	56.9 (51.8, 62.0)	169	46.6 (39.7, 53.6)
Somewhat agree	918	28.7 (26.7, 30.7)	194	33.8 (28.9, 38.7)	128	39.4 (32.3, 46.6)
Strongly/very strongly agree	146	5.4 (4.2, 6.5)	60	9.3 (6.5, 12.1)	47	14.0 (9.5, 18.4)
Adjusted prevalence difference (95% CI; q-value)§	Referent		3.4 (0.4, 6.4; 0.04)		7.9 (3.1, 12.6; 0.003)	
The United States needs a civil war to set things right.						
Do not agree	3017	86.3 (84.7, 88.0)	439	75.0 (70.5, 79.4)	237	69.9 (63.9, 75.9)
Somewhat agree	303	10.5 (9.0, 11.9)	102	17.9 (13.9, 21.9)	73	19.6 (14.7, 24.6)
Strongly/very strongly agree	84	3.2 (2.3, 4.1)	43	7.1 (4.6, 9.6)	34	10.5 (6.5, 14.5)
Adjusted prevalence difference (95% CI; q-value)§	Referent		3.7 (1.1, 6.3; 0.01)		6.9 (2.8, 11.0; 0.003)	

Details on classification of firearm owners by frequency of carrying are in the Supplemental Methods Text section of this supplement (p 13).

*Q: Some people talk about a second civil war in the United States. Which of the following comes closer to your view of what a second civil war might look like? R1: A second civil war would be like the first Civil War in the United States, with opposing armies and large battles. R2: A second civil war would be like an insurgency or guerrilla war, with small groups attacking specific targets or people.

† Response options were do not agree, somewhat agree, strongly agree, very strongly agree. Findings are combined for the strongly/very strongly agree responses.

§ Adjusted models include age, race and ethnicity, gender, education, income, Census division, and rurality. Adjusted differences are for the strongly/very strongly agree comparison. Q-values represent the probability that the given difference would be a false discovery; they represent

the expected proportion of “false positives” that would be seen among the collection of all differences whose q-values were at or below the given q-value.
